# Alzheimer's Disease and Frontotemporal Dementia: A Review of Pathophysiology and Therapeutic Approaches

**DOI:** 10.1002/jnr.70046

**Published:** 2025-05-19

**Authors:** Sally Kelliny, Xin‐Fu Zhou, Larisa Bobrovskaya

**Affiliations:** ^1^ Health and Biomedical Innovation, Clinical and Health Sciences University of South Australia Adelaide South Australia Australia; ^2^ Faculty of Pharmacy Assiut University Assiut Egypt

**Keywords:** amyloid beta, dementia, neurotrophins, oxidative stress, p75, tau

## Abstract

Alzheimer's disease (AD) is a devastating form of dementia, with the number of affected individuals rising sharply. The main hallmarks of the disease include amyloid‐beta plaque deposits and neurofibrillary tangles consisting of hyperphosphorylated tau protein, besides other pathological features that contribute to the disease's complexity. The causes of sporadic AD are multifactorial and mostly age‐related and involve risk factors such as diabetes and cardiovascular or cerebrovascular disorders. Frontotemporal dementia (FTD) is another type of dementia characterized by a spectrum of behaviors, memory, and motor abnormalities and associated with abnormal depositions of protein aggregation, including tau protein. Currently approved medications are symptomatic, and no disease‐modifying therapy is available to halt the disease progression. Therefore, the development of multi‐targeted therapeutic approaches could hold promise for the treatment of AD and other neurodegenerative disorders, including tauopathies. In this article, we will discuss the pathophysiology of AD and FTD, the proposed hypotheses, and current therapeutic approaches, highlighting the development of novel drug candidates and the progress of clinical trials in this field of research.

**Summary**

- This review discusses the hallmarks and pathophysiology of Alzheimer's disease (AD) and frontotemporal dementia (FTD).
- It covers current hypotheses while also highlighting new theories linking dementia to various underlying factors such as diabetes.
- Additionally, we overview some therapeutic strategies, including recent disease‐modifying treatments and current clinical trials.
- This is an in‐depth review of the biological mechanisms underlying dementia and the evolving approaches to treatment, offering valuable insights into how our understanding of these diseases is evolving and how the therapies could progress in the future.

## 1 Alzheimer's Disease and Frontotemporal Dementia: Epidemiology and Risk Factors

Alzheimer's disease (AD) is a severe neurodegenerative disorder characterized by gradual and progressive deficits in more than one domain of cognition, including learning and memory, attention, language, and deterioration of social skills (Braak et al. 1993). It accounts for about 80% of dementia cases and is considered the 7th leading cause of death in the United States (Alzheimer's Disease International 2018). AD is classified, based on the age of onset, into two categories: late‐onset AD (LOAD) or sporadic, which accounts for > 95% of AD cases, and early onset or familial (EOAD) which accounts for 1%–5% of AD cases (Reitz and Mayeux 2014). Familial or EOAD is mostly caused by genetic mutations in three autosomal dominant genes, which are amyloid‐β precursor protein (*APP*), presenilin‐1 (*PSEN1*) or presenilin‐2 (*PSEN2*). *APP* is the gene coding for the sequence of amyloid beta (Aβ), whereas *PSEN1* and *PSEN2* are involved in APP processing and Aβ generation (Campion et al. 1999). While sporadic AD, which accounts for most cases, is multifactorial, the exact causes of the disease are not yet recognized. Aging is considered the main risk factor. Other non‐genetic risk factors, including cardiovascular diseases (atherosclerosis), diabetes, lifestyle, cerebrovascular disease, and neuropsychiatric disorders (psychosis, depression) are comorbidities that are strongly associated with AD (Reitz and Mayeux 2014; Avitan et al. 2021). Apolipoprotein (apo) *E4*, referred to as the allele ε4 (*APOEε4*) gene, which encodes the ApoE4 lipid carrier (involved in Aβ binding and clearance), has been genetically linked to late‐onset AD. Carriers of one or two alleles of *APOε4* have ~3.5–10 times increased risk of developing the disease, respectively (Roses 2006; Aleshkov et al. 1997).

Other common causes of dementia, in addition to AD, include frontotemporal dementia (FTD) and Lewy body dementia. FTD is a spectrum of clinical syndromes characterized by a progressive decline in behavior and personality (the behavioral variant) and/or speech/language (the language variant; primary progressive aphasia (PPA)) with relative preservation of memory and with or without related features of motor neuron disease or parkinsonism (Sieben et al. 2012). It is associated with neurodegeneration of the frontal and anterior temporal lobes (Bott et al. 2014; Rademakers et al. 2012). FTD is considered the second most common cause of dementia in the younger population after AD and 3rd among the older population (Onyike and Diehl‐Schmid 2013). In FTD, 30%–50% of cases are familial and have a strong family history of dementia, while the remaining cases are sporadic (Panza et al. 2020). Familial cases are caused by gene mutations in Microtubule‐associated protein tau (*MAPT*) (Hutton et al. 1998), Chromosome 9 open reading frame 72 (*C9orf72*) (Snowden et al. 2012), Progranulin (*GRN*) (Pickering‐Brown et al. 2006), valosin‐containing protein (Watts et al. 2004) or Chromatin‐modifying protein 2B (Skibinski et al. 2005). 44 different mutations of tau on chromosome 17 have been identified in a number of families with frontotemporal dementia and parkinsonism (FTDP‐17) (Goedert and Jakes 2005). The Progranulin gene mutation was identified in 2006 as the second FTD‐related gene on chromosome 17 and is responsible for an even a larger proportion of FTD. Many fewer common mutations associated with the valosin‐containing protein gene cause a rare familial syndrome of Inclusion Body Myopathy with Paget disease of bone and FTD. Another rare mutation in the gene for Chromatin‐modifying protein 2B is found in a large Danish FTD lineage. A recent discovery of the mutation in the chromosome 9 open reading frame 72 has identified the most genetic cause of the FTD/amyotrophic lateral sclerosis (ALS) combination found in several families. Head trauma and thyroid disease were identified as non‐genetic risk factors of FTD and are associated with a 3.3‐fold and a 2.5‐fold higher risk, respectively (Rademakers et al. 2012; Onyike and Diehl‐Schmid 2013).

## 2 Neuropathological Hallmarks of AD and FTD

### 2.1 Neuropathological Hallmarks of AD and Mechanisms of the Disease

Alzheimer's disease is characterized by the accumulation of cerebral plaques loaded with β‐amyloid peptide (Aβ) and dystrophic neuritis distributed throughout the cortical layer, as well as intracellular neurofibrillary tangles composed of hyperphosphorylated tau protein in the medial temporal‐lobe structures (De Strooper and Karran 2016; Serrano‐Pozo et al. 2011). Extensive neuron loss, cerebral amyloid angiopathy (CAA), inflammation, synaptic failure, and oxidative damage are also core features of AD (Querfurth and LaFerla 2010). An early and massive loss of basal forebrain cholinergic neurons is also a key basis for cognitive impairment in AD, which has inspired the concept of the old cholinergic hypothesis of AD (Bartus et al. 1982).

#### 2.1.1 Amyloid Plaques

**FIGURE 1:**
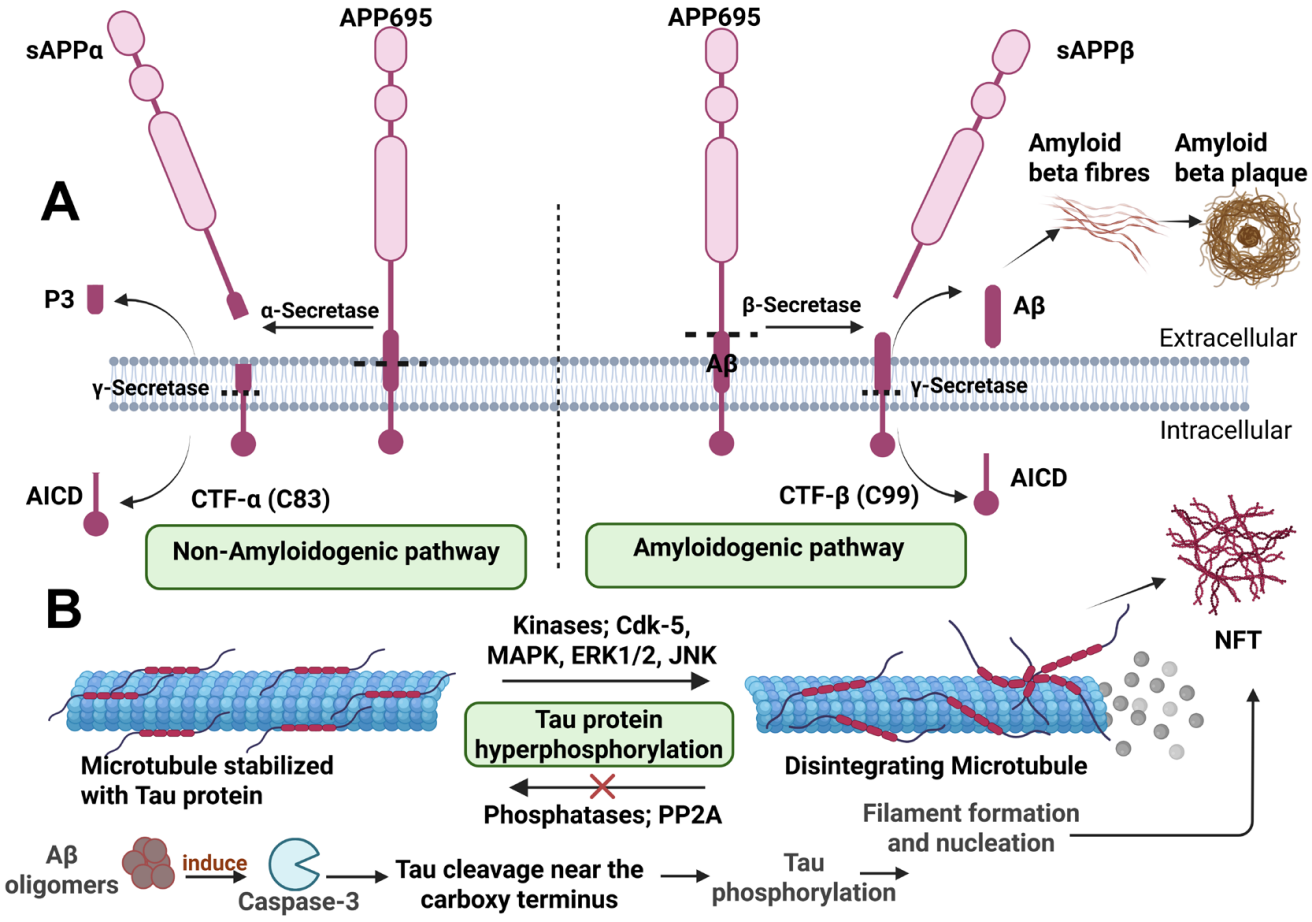
Mechanisms of formation of amyloid plaques and neurofibrillary tangles in AD. (A) The proteolytic cleavage of the amyloid precursor protein (APP) by the non‐amyloidogenic (left) and amyloidogenic (right) processing pathways. In the non‐amyloidogenic pathway, mature APP anchored to the plasma membrane can be cleaved by α‐secretase to release soluble APPα (sAPPα) and the C‐terminal fragment‐α (CTF‐α) which is further cleaved by γ‐secretase to generate the P3 peptide and the APP intracellular domain (AICD). In acidic environments such as endosomes, the amyloidogenic pathway (right) favorably occurs, in which reinternalized APP is consecutively processed by β‐secretase to produce soluble APPβ (sAPPβ) and the β‐cleaved carboxy‐terminal fragment (CTF‐β). γ‐Secretase then further processes CTF‐β into amyloid‐β (Aβ) and AICD. Aβ then spontaneously self‐aggregates into oligomers and other intermediate assemblies that can also grow into fibrils and self‐arrange into β‐pleated sheets to form the dense‐core plaques. (B) Tau mediated neurodegeneration. Cytoplasmic tau protein binds to tubulin during its polymerization and stabilizes microtubules. The attachment of tau to microtubules (MT) is regulated by its phosphorylation level. Tau phosphorylation is mediated by kinases such as mitogen‐activated protein kinase (MAPK), glycogen synthase kinase‐3 β (GSK‐3β) and c‐Jun N‐terminal kinase (JNK), and cyclin‐dependent kinase 5 (Cdk‐5) which play an important role in cytoskeletal organization. Dephosphorylation of tau is mediated by phosphatases such as PP2A that restore the binding ability of tau to MT. Under pathological conditions, such balance is disrupted due to activation of kinases and inhibition of phosphatases. This promotes tau hyperphosphorylation leading to its detachment from MT and MT depolymerization and disintegration. Hyperphosphorylated tau is misfolded and self‐assembled to paired helical filaments (PHF) and neurofibrillary tangles (NFT). Aβ can activate caspase‐3 that cleaves tau near its carboxy terminals and makes it prone to phosphorylation by kinases leading to tau nucleation and aggregation. The destabilization of MT and aggregation of NFT inside neurons disrupts axonal transport and synaptic plasticity, and finally induces cell loss.

Amyloid beta (Aβ) peptides, consisting of 36 to 43 amino acids, are naturally derived from amyloid precursor protein (APP), a type I transmembrane protein, after sequential cleavage by beta‐site amyloid precursor protein‐cleaving enzyme 1 (BACE‐1); β‐secretase, and γ‐secretase, which is a protein complex with presenilin 1 at its catalytic core (Haass and Selkoe 2007). The amyloidogenic pathway of APP is favored in acidic compartments such as endosomes, where reinternalized APP is first cleaved by β‐secretase to produce sAPPβ and 99‐residue carboxy‐terminal fragment (APP‐CT, C99), and that is further cleaved by γ‐secretase to generate Aβ and the APP intracellular domain (AICD). APP, anchored to the plasma membrane, may undergo non‐amyloidogenic cleavage by α‐secretase within the Aβ region, generating soluble sAPPα and C83, the latter of which is further processed by γ‐secretase to yield the P3 peptide and AICD (Zhang et al. 2019) (Figure 1A).

Aβ_40_ monomers are more dominant than the toxic and more hydrophobic Aβ_42_ species that shows a higher tendency of aggregation (Querfurth and LaFerla 2010). The exact function of Aβ is still controversial, but several reports suggested that at physiological levels, Aβ may play a role in modulation of synaptic plasticity (Morley et al. 2019; Bishop and Robinson 2004). An imbalance between production and clearance of Aβ can drive its aggregation, and this could be the initiating factor in AD. This theory is referred to as the “amyloid cascade hypothesis” that could explain the genetic forms of AD (Selkoe 2001) and the amyloid pathology in Down's syndrome (Busciglio et al. 2002). Aβ spontaneously self‐aggregates into soluble oligomers and other intermediate assemblies that are synaptotoxic, which can also grow into fibrils and self‐arrange into β‐pleated sheets to form the dense‐core plaques (primarily Aβ_42_) and CAA (mainly Aβ_40_) (Serrano‐Pozo et al. 2011; Querfurth and LaFerla 2010). The severity of cognitive impairment in AD patients correlates with the levels of the soluble oligomers in the brain, particularly Aβ_40_, rather than the total plaque load (Lue et al. 1999). Several lines of evidence indicate that increased levels of Aβ oligomers might initiate the synaptic damage and be responsible for neurodegeneration in AD (Selkoe 2008; Lacor et al. 2007; Glabe 2005; Townsend et al. 2006). Aβ is physiologically secreted from synapses, upon neuronal activation, in a process related to normal release of neurotransmitters (Querfurth and LaFerla 2010). In vitro studies showed that excessive Aβ release might dampen neuronal excitatory transmission (Kamenetz et al. 2003), damage synapses, and interfere with activity‐regulated cytoskeleton‐associated protein (Arc) distribution (Selkoe 2008). Therefore, synaptic function is the best indicator of the extent of cognitive impairment in AD patients and is well correlated with the levels of cerebral Aβ oligomers.

Steady‐state levels of Aβ are regulated by proteases neprilysin and insulin‐degrading enzyme (IDE). Neprilysin (NEP) is a membrane‐anchored zinc endopeptidase that is capable of degrading Aβ monomers as well as oligomers (Kanemitsu et al. 2003), while insulin‐degrading enzyme, a thiol metalloendopeptidase, degrades small peptides such as insulin and Aβ monomers (Qiu et al. 1998). Animal studies showed that overexpression of these enzymes prevents plaque formation (Leissring et al. 2003). In contrast, deletion of IDE or reduction of neprilysin reduces Aβ degradation by more than 50% and drives its accumulation (Iwata et al. 2001; Farris et al. 2003). However, analysis of NEP and IDE levels and activity in the post‐mortem frontal cortex from sporadic AD patients at different ages showed that reduction of NEP and IDE is not a primary cause of Aβ accumulation, but these changes only occur in late stages of the disease secondary to neurodegeneration (Miners et al. 2009).

#### 2.1.2 Cerebral Amyloid Angiopathy (CAA)

CAA is caused by deposits of Aβ (mainly Aβ_40_) in the arteries and cortical capillaries, especially in the posterior parts of the brain that weaken the vessel wall and can lead to life‐threatening lobar hemorrhages (Serrano‐Pozo et al. 2011).

#### 2.1.3 Neurofibrillary Tangles (NFTs)

NFTs are filamentous inclusions of hyperphosphorylated and misfolded tau protein within the pyramidal neurons and occur in AD and tauopathies (Querfurth and LaFerla 2010; Götz et al. 2019). NFTs are accompanied by neuropil threads (NT) which result from the breakdown of dendrites and axons of the tangle‐bearing neurons (Serrano‐Pozo et al. 2011). In contrast to amyloid deposits, NFTs exhibit a characteristic distribution pattern and spatiotemporal progression that correlates with the severity and duration of AD (Serrano‐Pozo et al. 2011; Arriagada et al. 1992; Ingelsson et al. 2004; Giannakopoulos et al. 2003). In 1991, Braak H. and Braak E. developed a qualitative evaluation method that allowed the differentiation of neuropathological stages of AD based on the changes in the distribution pattern of NFT and NT (Braak and Braak 1991).

Tau is a highly soluble cytoplasmic protein that promotes axonal transport through binding to tubulin during its polymerization onto microtubules, promoting its assembly and stabilization. Tau also functions in neurite outgrowth and acts as a postsynaptic scaffolding protein, modulating the activity of Src tyrosine kinases in actin remodeling (Sharma et al. 2007; Mietelska‐Porowska et al. 2014) and facilitating the postsynaptic trafficking of Fyn to PSD‐95 (Postsynaptic density protein‐95)/NMDA (N‐methyl‐d‐aspartate) glutamate receptors signaling complex (Ittner et al. 2010). In pathologic conditions, tau undergoes aberrant phosphorylation and misfolding that leads to its dissociation from microtubules and makes it prone to self‐assembly and aggregation to paired helical filaments (PHF) and NFT (Figure 1B). Accumulation of tau causes disruption of the cytoskeleton and axonal transport, interferes with mitochondrial dynamics (DuBoff et al. 2012), and makes neurons more susceptible to oxidative stress (OS) damage, thereby accelerating degeneration (Stamer et al. 2002). Similar to Aβ oligomers, intermediate tau aggregates are cytotoxic and impair cognition because they can bind to healthy tau, promoting its detachment from microtubules (Oddo et al. 2006). In contrast, NFT filaments are believed to be the inert form that the neurons are packaging tau into as a protective response to sequester the toxic intermediate tau species (Iqbal et al. 2005; Lee et al. 2005). What controls the extent of tau phosphorylation is the balance between the activity of tau kinases and phosphatases, mainly protein phosphatase 2A (PP2A), as well as tau conformation that affects its interaction with these enzymes (Iqbal et al. 2005). Glycogen synthase kinase (GSK‐3), cyclin‐dependent protein kinase‐5 (Cdk‐5), protein kinase A (PKA), mitogen‐activated protein (MAP) kinase ERK1/2, and stress‐activated protein kinases (C‐Jun amino‐terminal kinase (JNK)) are the main kinases implicated in abnormal tau phosphorylation (Iqbal et al. 2005). Studies also showed that caspase‐mediated cleavage of tau at the carboxy‐terminus, likely by caspase‐3 (Gamblin et al. 2003), alters tau conformation, facilitating its hyperphosphorylation and fibrillization, and hence is considered a key pathway in tangle formation (Guillozet‐Bongaarts et al. 2005; Rissman et al. 2004; Cotman et al. 2005). Interestingly, studies showed that Aβ induces caspase activation and the subsequent truncation of Tau (Gamblin et al. 2003; Rissman et al. 2004) in addition to activating GSK‐3 and Cdk tau kinases (Terwel et al. 2008; Zheng et al. 2002; Kim et al. 2003; Hoshi et al. 2003; Liu et al. 2004), thus driving tau pathology. Evidence from in vitro and in vivo experimental studies indicated that Aβ precedes and drives tau aggregation (Oddo et al. 2003; Götz et al. 2001; Lewis et al. 2001). Furthermore, it was shown that tau is necessary for Aβ‐induced neurotoxicity in cultured hippocampal neurons and cognitive deficits in AD mice, and that targeting tau pathology could block Aβ‐induced neurotoxicity (Roberson et al. 2007; Rapoport et al. 2002). These findings provide a mechanistic link between Aβ and tau in AD. In addition, OS, impaired protein‐folding function of the endoplasmic reticulum, and deficient proteasome‐mediated and autophagic‐mediated clearance of damaged proteins are all associated with aging and accelerate the accumulation of amyloid and tau proteins in AD (Hoozemans et al. 2005; López Salon et al. 2000).

It is worth mentioning that some patients with no overt symptoms of dementia antemortem show profuse Aβ plaques in the brain similar to AD patients (referred to as high pathology controls). But levels of soluble Aβ, the presence of CAA, NFT, and neuropil threads surrounding the amyloid core, besides inflammation, could only distinguish high pathology controls from AD patients (Lue et al. 1999, 1996).

#### 2.1.4 Synaptic Failure

AD is primarily identified as a disorder of synaptic failure (Querfurth and LaFerla 2010; Selkoe 2002). Synapse loss contributes to the cortical atrophy of the AD brain (Serrano‐Pozo et al. 2011) and is the best correlate with the degree of dementia (DeKosky and Scheff 1990). Hippocampal synapses start to decline in patients with mild cognitive impairment (MCI), while their remaining synapses increase in size (become enlarged) as a compensation for reduced synaptic density/number (Scheff et al. 2007). The expression levels of the presynaptic protein synaptophysin are reduced by 25% in mild (early) AD (Masliah et al. 2001). With disease progression, synaptic loss can surpass neuron loss, and both synapse number and size decline (DeKosky and Scheff 1990).

Aging itself induces synaptic failure (Masliah et al. 2006). Intraneuronal soluble Aβ oligomers can also trigger neuronal injury and synaptic deficits. Chen et al. showed that applying Aβ_42_ peptides to rat hippocampal slices inhibited long‐term potentiation (LTP) through calcineurin‐dependent mechanisms, shifting the balance towards depression (Chen et al. 2002). Aβ‐induced spine loss is believed to be mediated through acting on NMDA glutamate receptors (NMDAR), causing disruption of calcium influx, thus mimicking a state of partial NMDAR blockage (Lue et al. 1999; Shankar et al. 2007). This induces LTD that is associated with synaptic shrinkage and retraction (Zhou et al. 2004). Aβ also triggers endocytosis of NMDA (Snyder et al. 2005) and postsynaptic AMPA (α‐amino‐3‐hydroxy‐5‐methyl‐4‐isoxazole propionic acid) glutamate surface receptors (Hsieh et al. 2006), the latter of which induces a prolonged depression following LTP‐inducing stimulus train. Similar to Aβ, aging impairs basal synaptic transmission and induces NMDAR‐dependent inhibition of LTP by disrupting intracellular Ca^2+^ signaling (Foster and Norris 1997). Therefore, it is tempting to say that Aβ may exacerbate aging‐related Ca^2+^ dysregulation and synaptic dysfunction (Chen et al. 2002). Moreover, Aβ also binds to p75 neurotrophin receptor and tyrosine kinase B receptor (TrkB, a receptor for brain‐derived neurotrophic factor (BDNF)) in synapse terminals, provoking a situation in which the levels of BDNF and nerve growth factor (NGF) are already suppressed while the levels of proneurotrophins are increased, leading to neurodegeneration. Aβ may also bind to nicotinic acetylcholine receptors (nAChR), suppressing cholinergic transmission and the ACh (acetylcholine) release from the presynaptic terminal (Querfurth and LaFerla 2010).

Evidence indicates that Aβ_42_ may disturb intracellular calcium homeostasis by forming ion channel‐like structures in the lipid bilayer membrane of Neuro‐2a (N2A) mouse neuroblastoma cells, causing an increase in calcium influx and intraneuronal calcium levels, thereby activating signal transduction pathways leading to neurotoxicity and degeneration (Lin et al. 2001).

On the other hand, accumulation of phosphorylated tau at synaptic terminals and its relocation to dendritic spines is also synaptotoxic. It may interact with the PSD‐95/NMDAR complex and enhance calcium influx and may impair the synaptic trafficking and anchoring of glutamate receptors (Hoover et al. 2010). It may lead to tau oligomer deposition and disruption of the synaptic ubiquitin‐proteasome system (Tai et al. 2012). Also, misfolded tau may propagate to neighboring neurons along synaptic networks (Liu et al. 2012; Dujardin et al. 2014; de Calignon et al. 2012; Ahmed et al. 2014) via “prion‐like” and seeding mechanisms (Walker et al. 2013), and such transmission is accelerated by Aβ (Pooler et al. 2015).

#### 2.1.5 Neuroinflammation

Neuritic plaques, NFTs, and injured neurons may all provoke inflammation in the AD brain. Activated microglia and astrocytes cluster in the vicinity of neuritic plaques, and their biochemical markers are elevated in the brains of AD patients (Wyss‐Coray and Mucke 2002). Aging can augment the inflammatory response in the brain and render it vulnerable to Aβ neurotoxicity (Nichols et al. 1993; Geula et al. 1998). Streit et al. first demonstrated that aged and AD brains show senescent and dystrophic microglia and suggested that these morphological changes make them dysfunctional and unable to clear Aβ (Streit et al. 2004). Aβ is one of the potent triggers for inflammation in AD (Moore and O'Banion 2002). In vitro studies demonstrated that Aβ and APP could initiate inflammation by inducing glia activation and the subsequent release of a variety of inflammatory mediators, such as interleukin‐1β (IL‐1β) and tumor necrosis factor‐α (TNF‐α), as well as free radicals, such as nitric oxide (NO) (Barger and Harmon 1997). The release of such proteins and oxidants enhances Aβ‐induced glial activation (Hu and Van Eldik 1999), amplifies the inflammatory response, and inhibits the microglial phagocytosis of Aβ peptides (Webster et al. 2000). Therefore, this state of chronic activation creates a continuous cycle of detrimental inflammatory stimuli that lead to neurotoxicity (Moore and O'Banion 2002; Akama et al. 1998). A hyperactive microglial state, combined with ineffective phagocytic ability, may lead to aggregation of Aβ from diffuse to neuritic plaques and inefficient clearance of damaged neurites, creating a focal spot for the accumulation of aggregation‐prone peptides. Cell culture studies revealed that Aβ could stimulate glia by activating NF‐κB (nuclear factor kappa B), a transcription factor implicated in the induction of genes, including cytokines and immunoreceptors (Akama et al. 1998). Activation of NF‐κB by Aβ possibly occurs via binding to RAGE, the receptor for advanced glycation end products (Lue et al. 2001), or through Aβ's chemokine‐like activity acting at formyl chemotactic receptors (Lorton et al. 2000).

#### 2.1.6 Mitochondrial Dysfunction and Oxidative Stress (OS)

Mitochondrial dysfunction and OS have been associated with the pathogenesis of AD and occur early prior to the appearance of plaque pathology (Manczak et al. 2006; Maurer et al. 2000; Subbarao et al. 1990). Aβ itself is a mitochondrial poison. Several studies showed that Aβ and APP may accumulate inside the mitochondria (Mungarro‐Menchaca et al. 2002) and across its import channels (Devi et al. 2006), causing morphological changes, including swelling and disruption of its function. Aβ may inhibit the respiratory chain complex IV (cytochrome *c* oxidase, COX) and key Krebs‐cycle enzymes (α‐ketoglutarate and pyruvate dehydrogenase), leading to impaired oxygen consumption and energy production and resulting in the generation of free radicals and reactive oxygen species (ROS) (Caspersen et al. 2005). Aβ may also induce the loss of mitochondrial membrane potential and the opening of the permeability‐transition pores (ψm) that is exacerbated by the presence of pathogenic tau (Pallo and Johnson 2015). These defects could result in mitochondrial fragmentation (Barsoum et al. 2006), provoke OS, and trigger apoptotic signaling pathways (Mattson et al. 1998). In fact, AD and normal aging brains gradually accumulate mitochondrial DNA mutations due to sustaining high levels of oxidative damage, which likely contribute to mitochondrial defects in AD (Coskun et al. 2004).

In addition, compiling evidence suggests a possible role of tau in mitochondrial dysfunction and OS. Transgenic mice bearing the human pathogenic mutation P301L of tau, a model of frontotemporal dementia with parkinsonism‐17 (FTDP‐17), exhibited mitochondrial dysfunction and impaired energy production associated with the accumulation of ROS with aging. Phosphorylated tau specifically affects the activity of complex I (Mondragón‐Rodríguez et al. 2013) and hinders axonal trafficking of mitochondria and their synaptic docking due to microtubule destabilization (Tramutola et al. 2017; Tönnies and Trushina 2017). Mitochondrial dysfunction can also cause tau hyperphosphorylation. Besides, P301L tau mitochondria were vulnerable to Aβ‐induced neurotoxic insult. These findings suggest a synergistic action of tau and Aβ pathology on mitochondrial dysfunction, OS, and synaptic plasticity (David et al. 2005).

Dysfunctional mitochondria in AD are the major source of OS. Advanced glycation end products (AGEs) formed from non‐enzymatic reactions of sugar ketones or aldehydes with protein can induce OS by acting on the RAGE receptor, in the same way as Aβ, modulating gene transcription involved in inflammation through NF‐κB. Accumulation of AGEs is a feature of aging (Srikanth et al. 2011) and age‐related diseases such as diabetes mellitus (Münch et al. 1998). Also, stimulated microglia release reactive oxygen (ROS)/reactive nitrogen species (RNS) as a response mechanism for attacking opsonized targets. These oxidants bind with membrane lipids and several proteins, inducing damage to several molecular targets (Su et al. 2008).

OS can upregulate tau phosphorylation in AD through activation of kinases such as GSK3β and mitogen‐activated protein kinases (MAPKs) (Zhu, Castellani, et al. 2001), including JNK, a stress‐activated protein kinase (SAPK) that amplifies stress signals to the nucleus (Zhu, Raina, et al. 2001). Oxidants such as 4‐hydroxy‐2‐nonenal (4‐HNE) and carbonyls modify tau protein, rendering it more prone to aggregate and polymerize (Pérez et al. 2000). SAPK/JNK induced by OS is also involved in Aβ deposition through provoking APP phosphorylation at Threonine 668, favoring its amyloidogenic processing (Colombo et al. 2009; Savage et al. 2002). OS induced by hydrogen peroxide may potentiate BACE1 gene expression and activity and enhance Aβ formation (Tong et al. 2005). In turn, Aβ may trigger JNK activation, leading to neuron death (Shoji et al. 2000). Therefore, it is suggested that OS plays a major role in inducing tau and Aβ accumulation in AD through upregulation of upstream signaling pathways, while Aβ and tau aggravate the inflammatory and OS cellular responses in a positive feedback loop leading to neurotoxicity (Su et al. 2008). These findings strongly suggest that targeting OS and the mitochondria could be a successful strategy for AD therapeutics.

#### 2.1.7 Neuronal Loss

Massive neuronal loss is a main pathological feature in the hippocampus and cerebral cortex of AD patients. Evidence of neuronal apoptosis is observed in sporadic and familial AD brains. Collapse of the mitochondrial membrane potential and opening of the permeability‐transition pores activates caspase‐3, initiating cell lysis and proteolysis. In addition, Aβ is a key mediator of degeneration not only in neurons but also in cerebral endothelial cells and astrocytes that constitute the blood‐brain barrier (BBB) (Hsu et al. 2007). Aβ activates stress‐activated protein kinases p38 MAPK and JNK/c‐Jun pathways as well as the mitochondrial regulatory protein p53 and triggers apoptotic signaling through p53 upregulated‐modulator of apoptosis (PUMA), proapoptotic protein Bim (Bcl‐2 interacting mediator of cell death), and Bax (Querfurth and LaFerla 2010; Akhter et al. 2015). Furthermore, in vitro studies showed that Aβ triggers a rapid and sustained downregulation of a key antiapoptotic protein, Bcl‐2, in human neuron primary cultures, while it increases levels of Bax; therefore, enhancing neuron vulnerability to age‐related stresses such as OS (Paradis et al. 1996). Interestingly, Tamagno et al. showed that all these biological events induced by Aβ were stimulated by OS reactive intermediates and were significantly prevented or blocked when cells were pre‐treated with antioxidants or by specific SAPKs inhibitors, suggesting a direct involvement of OS in Aβ‐induced apoptotic signaling (Tamagno et al. 2003).

### 2.2 Genetics and Neuropathology of FTD

In FTD, sometimes referred to as frontotemporal lobar degeneration (FTLD), astrogliosis and neuron loss are observed in the cortices of the affected frontal and temporal lobes. OS is another component of the pathology of FTD, especially in subtypes associated with tau accumulation. FTLD is characterized by intracellular accumulation of abnormal protein inclusion in the cytoplasm or nuclei of neuronal and glial cells. Based on the constituents of these inclusions, FTLD was divided into subcategories (Morris et al. 2001; Mackenzie, Neumann, et al. 2010). Initially, the first group was identified by the accumulation of hyperphosphorylated tau protein and referred to as FTLD‐tau, including Pick's disease (~40% of FTD cases) (Mackenzie, Rademakers, et al. 2010). Later, another type of inclusion, found in > 50% of patients, is tau‐negative and ubiquitin‐positive. These inclusions were recognized later to be formed of transactive response (TAR) DNA‐binding protein‐43 (TDP‐43), thus referred to as FTLD‐TDP (Neumann et al. 2006; Lipton et al. 2004). The majority of the remaining cases are TDP43‐negative cases and are associated with inclusions of fused‐in‐sarcoma protein (FUS), thus referred to as FTLD‐FUS, which is the cause of familial amyotrophic lateral sclerosis (ALS) type 6 (Mackenzie, Neumann, et al. 2010). Yet, some patients show protein inclusions that remain unknown to this day, referred to as FTLD‐ubiquitin‐proteasome system (FTLD‐UPS) (Sieben et al. 2012; Dickson and R 2011). In the following few sentences, we will focus on mutations in the MAPT gene, the Tau gene, that account for 5% of FTD cases and are frequently associated with parkinsonism (FTDP‐17).

**FIGURE 2:**
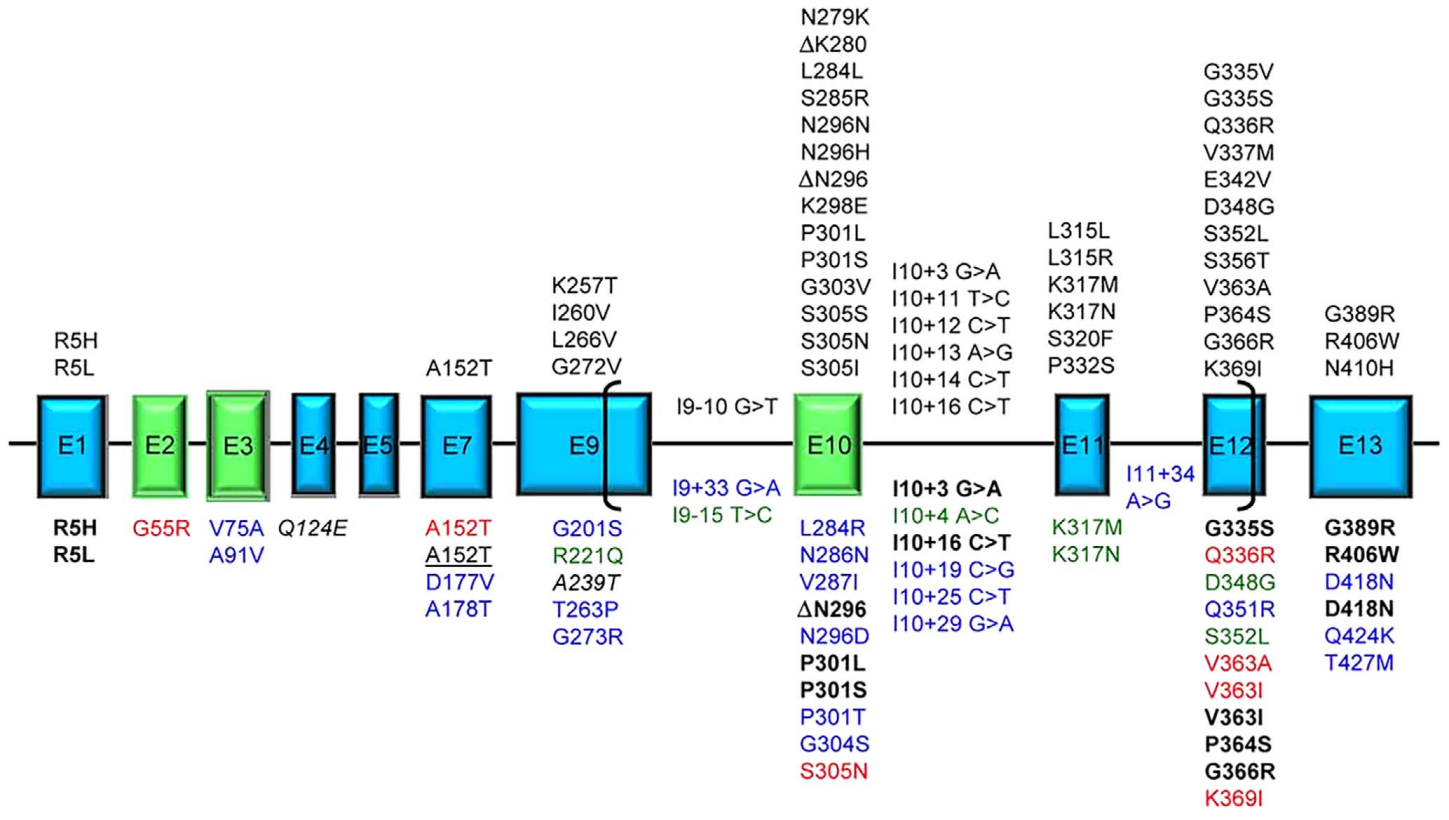
Tau mutations in frontotemporal lobar degeneration. Exons expressed in central nervous system (CNS), producing the longest 441 aa 2N4R tau isoform, are represented as boxes roughly proportional to their relative sizes; introns are represented as horizontal lines and are not proportional to their sizes. Exons subjected to alternative splicing are in green. Brackets include the region of microtubule‐binding domain (MBD). Above the exon boxes are the reported pathogenic mutations by genetic, neuropathological or functional evidence [reducing microtubule (MT) polymerization or increasing fibril formation]. Below the exon boxes, the mutations are classified by different colors: Bold black: Displaying unusual pathogenetic mechanisms; red: Affecting MT polymerization or fibril formation in uncommon ways; blue: Uncharacterized found in frontotemporal lobar degeneration (FTLD) or Alzheimer's disease (AD) patients; green: Atypical by clinical, neuropathological or transmission pattern features; italic: Risk factors for tauopathy when associated with mutations in other genes; underlined: Risk factors for tauopathy (FTLD or AD). A single mutation can have different features and be reported more than once in the figure. Reproduced with permission from Rossi and Tagliavini (2015).

Several studies showed that MAPT mutations, including R5L, K257T, I260V, G272V, DK280, P301L, P301S, Q336R, V337M, and R406W, may promote tau filament formation, reduce binding of mutant tau to protein phosphatase 2A (PP2A), reduce tau binding to microtubules, and enhance tau hyperphosphorylation (Goedert and Jakes 2005). These pathogenic tau mutations may either affect mRNA splicing of exon 10, or they are missense mutations located inside or outside exon 10. Most coding region mutations in exon 10 (N279K, L284L, DN296, N296N, N296H, S305N, and S305S) enhance splicing of exon 10, thus shifting the ratio between three‐ and four‐repeat isoforms. These mutations lead to the formation of wide, twisted, ribbon‐like filaments that consist only of four‐repeat tau isoforms (Jiang et al. 2003). On the other hand, some tau mutations on exon 10 do not affect mRNA splicing, such as the P301L mutation, which is relatively common in FTDP‐17 families. Isolated tau filaments from patients are of narrow, twisted, ribbon‐like morphology, predominantly consisting of four‐repeat mutant tau isoforms. The aforementioned tau mutations are widespread in both nerve cells and glial cells (Mirra et al. 1999). This contrasts with AD, in which paired helical tau filaments (PHF) are only present within nerve cells and consist of all six tau isoforms (Goedert and Spillantini 2011). Other mutations were found in exons 9–13, with the resultant tau pathology varying in filamentous morphology and isoform composition according to the position and the nature of the mutation and resembling that of sporadic tauopathies such as progressive supranuclear palsy (PSP), corticobasal degeneration (CBD), AD, or Pick's disease (PiD) (Goedert and Jakes 2005) (Figure 2).

## 3 AD Hypotheses

The exact mechanism of AD is yet to be fully understood. However, neuroinflammation, OS, and neurotrophic imbalance that can occur during aging are thought to be the major contributing mechanisms that lead to sporadic AD. Some of these mechanisms are covered below.

### 3.1 Amyloid Cascade Hypothesis

**FIGURE 3:**
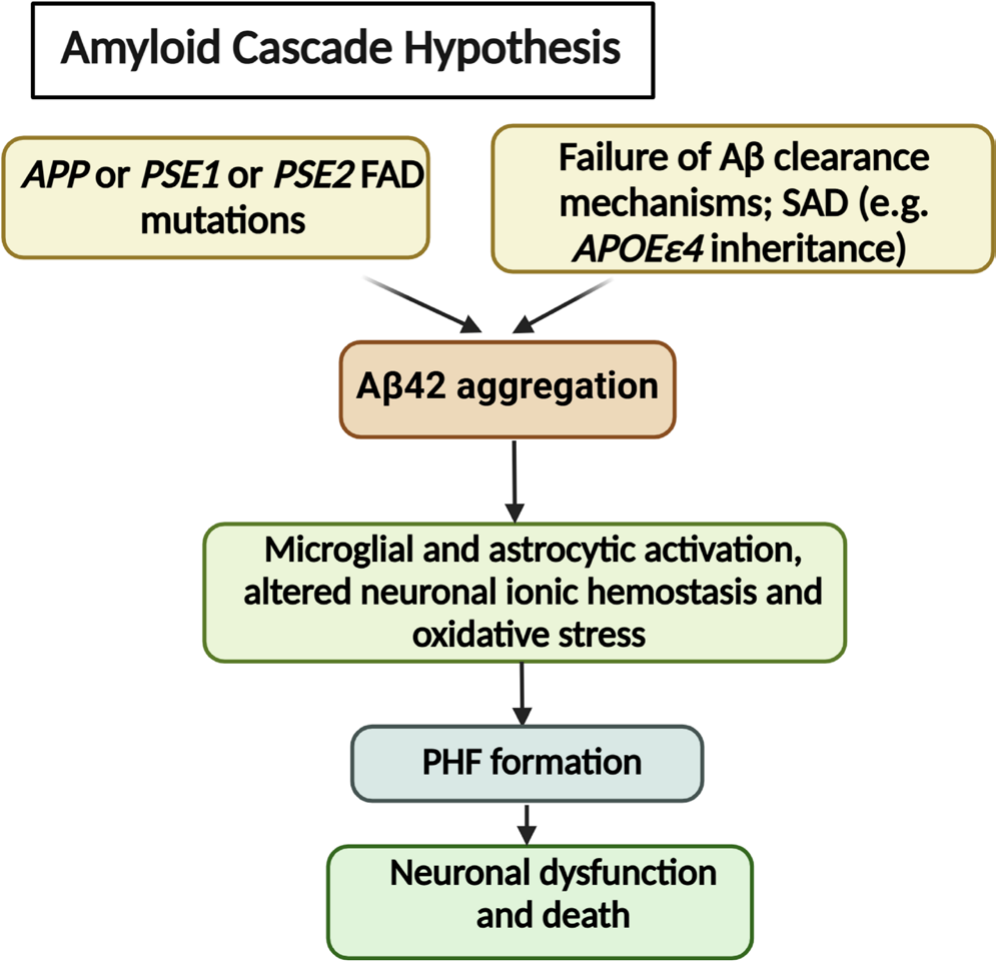
Overview of the amyloid cascade hypothesis. The imbalance between Aβ production and clearance due to certain inherited genetic mutations leads to the accumulation of amyloid beta aggregates, and these aggregates then initiate a cascade of events including microglial and astrocyte activation, oxidative stress, and tau accumulation that eventually lead to neuronal dysfunction and death (Selkoe and Hardy 2016). APOEε4, apolipoprotein (apo) E4, referred to as allele ε4; APP, amyloid precursor protein; FAD, familial AD; PHF, paired helical filaments; PSE, presenilin; SAD, sporadic AD.

The amyloid cascade hypothesis, postulated in 1992 by Hardy and Higgins, assumes that “Aβ disposition is a central event and causative agent of the AD pathogenic cascade and that tau tangles, cell loss, vascular damage, and dementia follow as a direct result of this disposition” (Hardy and Allsop 1991; Hardy and Higgins 1992). It combines histopathological and genetic material and suggests that the deposition of the Aβ peptides in the brain parenchyma, due to imbalance between Aβ production and clearance (Selkoe and Hardy 2016), initiates a series of events that eventually lead to AD dementia (Karran et al. 2011) (Figure 3). Support for this hypothesis came from autosomal‐dominant familial AD cases, which ultimately originate from aberrant Aβ production and aggregation due to *APP*, *PSEN1*, or *PSEN2* gene mutations (Sherrington et al. 1995; Levy‐Lahad et al. 1995; Tomiyama et al. 2008) or due to defects in Aβ clearance as a result of the presence of the *APOE4* allele in late‐onset AD (Holtzman et al. 2000; Kim et al. 2009). However, therapeutic approaches centered on Aβ have failed in phase III clinical trials. Also, it becomes evident that there is no linear correlation between cognitive alterations in dementia and Aβ plaques in humans. In particular, this hypothesis did not explain the driving force for amyloidosis in sporadic AD, which is actually a consequence of aging and not linked to genetic risk factors. This calls upon reconsidering and modifying the amyloid cascade hypothesis. However, Selkoe and Hardy believe that the discovery of various genetic risk loci for late‐onset AD through the genome‐wide association (GWA) studies, such as *TREM2* and *CD33*, although very rare, could support the amyloid cascade hypothesis (Selkoe and Hardy 2016). Such risk genes are involved in processes regulating cholesterol/sterol metabolism, modulating the inflammatory response of microglia to Aβ and the brain's innate immune system, and endosomal vesicle recycling. These processes might be addressed with available drugs such as statins or anti‐inflammatory agents (Jones et al. 2010; Sala Frigerio et al. 2019).

Also, this theory could not describe the relationship between Aβ and tau (Karran et al. 2011; Price et al. 2009). In sporadic AD, tau pathology may precede amyloid beta (Braak et al. 2013; Arnsten et al. 2021). In sporadic AD, Aβ and tau may evolve separately; however, both interact reciprocally and synergistically in mediating neurodegeneration (Park and Ferreira 2005; Mark et al. 2002). While Aβ is essential to trigger tau pathology, it does not correlate with cell loss or dementia. Aβ plaque pathology starts to appear in regions such as the precuneus and frontal lobes that are anatomically disconnected from those showing extensive neuron loss (hippocampus and entorhinal cortex). This is opposite to tau, which follows a regular spatiotemporal progression that clearly correlates with neuronal loss, cerebral atrophy, and dementia (Karran et al. 2011). It is thus assumed that Aβ initiates a neurotoxic complex process through inducing early tau phosphorylation; then tau mediates the downstream signaling of degeneration (Amadoro et al. 2011; Wu et al. 2018). In other words, tau is essential for Aβ‐induced neurotoxicity (Rapoport et al. 2002). A scenario by Karran et al. suggested that once this pathogenic cascade starts, it becomes self‐sustaining, more complex, and irrelevant to Aβ (Karran et al. 2011). Therefore, Aβ‐targeted therapeutic approaches could be beneficial, provided they are administered early in the disease course. This conclusion was supported by findings from clinical trials of Aβ antibodies showing that they have slowed the cognitive decline in mild AD patients but showed no benefit in moderate cases while their brains still showed heavy distribution of tau pathology across the cortex (Selkoe and Hardy 2016). In line with these findings, a study found that suppression of endogenous wild‐type tau prevented cognitive deficits in an AD mouse model expressing human mutant APP without detectable changes in plaque pathology (Roberson et al. 2007). Also, targeting both Aβ and tau, not only Aβ, by passive immunotherapy was able to ameliorate cognitive deficits in a 3×Tg AD animal model (Oddo et al. 2006, 2004). In addition, GSK‐3β inhibitors reduced Aβ‐induced cell death in hippocampal neurons following NGF withdrawal (Amadoro et al. 2011).

The amyloid cascade hypothesis was further modified to point out the early and toxic effects of Aβ oligomers (Benilova et al. 2012) and considers synapses as early targets for these toxic species (Selkoe 2002). However, the negative results and adverse effects of Aβ immunization in clinical studies called for reconsidering the whole hypothesis.

In 2008, Small and Duff hypothesized that “common upstream drivers cause both elevation in Aβ and tau hyperphosphorylation through independent but parallel mechanisms”. This hypothesis is referred to as “dual hit”, and the authors believe that this theory might explain the molecular mechanisms in late‐onset AD (Small and Duff 2008). But this theory again failed to explain the relationship between APP/Aβ and tau.

Musiek and Holtzman (2015) assumed that Aβ is likely a key initiator of a complex pathogenic cascade which causes AD, thus supporting the amyloid cascade hypothesis in general. However, Aβ may not act chiefly to trigger other downstream processes. Thus, Aβ seems to be necessary but not sufficient to cause AD. Instead, a complex network of pathologic processes, including age‐related stressors such as OS, congregate to produce the neuropathologic and clinical features of AD.

### 3.2 Tau Hypothesis

**FIGURE 4:**
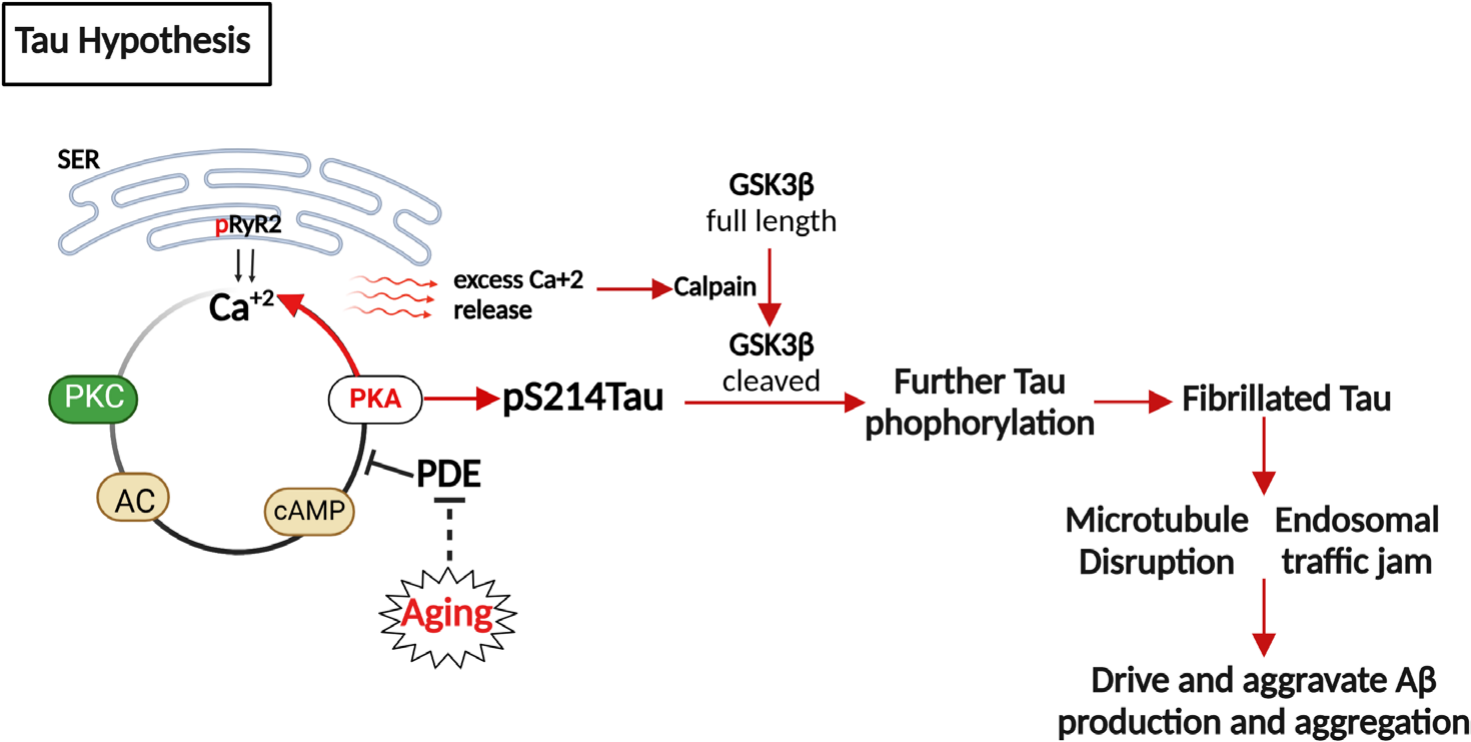
Overview of the tau hypothesis and the role of calcium dysregulation in tau pathology. Calcium release near the synapse from the smooth endoplasmic reticulum (SER) through calcium channel ryanodine receptors (RyR2), is stimulated by cyclic adenosine monophosphate (cAMP)‐ protein kinase A (PKA) signaling calcium release, a process that is controlled by phosphodiesterase (PDE4), which catabolizes cAMP. However, with aging, PDE4 activity is lost, leading to increased PKA activity that phosphorylates ryanodine receptors (pS2808RyR2), causing calcium leakage into the cytosol. Increased PKA activity also phosphorylates tau at S214,17, priming tau for hyperphosphorylation by the kinase GSK3β. PKA inhibits full‐length GSK3β when cytosolic calcium levels are normal. However, sufficiently high levels of calcium activate the protease calpain that can cleave GSK3β at the N‐terminus, removing its inhibition by PKA and switching the system into a later stage of tau hyperphosphorylation.

Recent reports suggest that tau may be the initiating factor in sporadic AD, as early tau inclusion may develop decades before Aβ plaques, according to Braak staging (Braak et al. 2011). It is hypothesized that calcium dysregulation may drive tau phosphorylation in aging due to the loss of phosphodiesterase (PDE4) activity, which catabolizes cyclic adenosine monophosphate (cAMP). This results in the activation of cAMP/protein kinase A (PKA) signaling that causes calcium leakage to the cytosol and primes tau for subsequent phosphorylation by GSK3β. Also, it is suggested that tau fibrils may trap endosomes containing APP and increase APP and BACE accumulation by disrupting microtubule transport, thus leading to the amplification of Aβ production. This may create a vicious cycle where pathologic tau induces Aβ production, and Aβ then induces further tau phosphorylation (Arnsten et al. 2021) (Figure 4). However, some argue against this hypothesis, referring to FTD‐tau cases that do not have amyloid plaque pathology.

### 3.3 Neurotrophin Hypothesis

According to the neurotrophin hypothesis, the imbalance of neurotrophins and their receptors during aging plays a critical role in abnormal Aβ accumulation, cholinergic and synaptic dysfunction, and hence, neurodegeneration in sporadic AD (Zhou 2016; Costantini et al. 2006; Cattaneo and Calissano 2012). Cattaneo's group was the first to provide evidence that neurotrophic deficits are the upstream driver by developing comprehensive AD‐like neurodegeneration in anti‐NGF ad11 mice (Cattaneo and Calissano 2012; Capsoni et al. 2000), which was reversed by early NGF delivery through an olfactory route (Capsoni et al. 2002). An earlier study by Costantini et al. (Costantini et al. 2005) suggested that the expression of the TrkA tyrosine kinase receptor predominates in younger age, mediating neuron survival. While during aging, a switch from TrkA to p75 neurotrophin receptor (p75^NTR^), a death receptor, occurs that promotes APP processing, stabilizes BACE1 expression, and enhances Aβ production. In a positive feedback loop, Aβ binds to p75^NTR^ with high affinity and activates receptor signaling, including MAPKs, JNK, p53, and NF‐κB translocation, leading to caspase‐3 activation and apoptotic cell death. Therefore, p75^NTR^ plays a crucial role in Aβ accumulation and in mediating Aβ‐induced neurotoxicity in aging (Costantini et al. 2005; Knowles et al. 2009; Sotthibundhu et al. 2008; Yaar et al. 1997, 2002).

#### 3.3.1 Neurotrophin Signaling

Neurotrophins, including BDNF, NGF, neurotrophin‐3 (NT‐3), and NT‐4/5, are trophic factors known to regulate several aspects of neuronal functions during development and in the adult nervous system by maintaining neuron survival, differentiation, axonal growth, myelination, expression of ion channels and neurotransmitters, and synaptogenesis (Chao 2003). They have been implicated in the pathophysiology of several neurodegenerative and neuropsychiatric disorders such as AD and depression. Neurotrophins activate two different types of receptors: the Trk (tropomyosin‐related kinase), a tyrosine kinase receptor family, and p75^NTR^, a 75kDa type I transmembrane protein that belongs to the TNF (tumor necrosis factor) receptor superfamily together with sortilin, which is a neuronal type‐1 VPS10‐domain receptor, a Golgi sorting protein, and the co‐receptor with p75^NTR^ for proneurotrophins. Through Trks and p75^NTR^, neurotrophins activate many signaling pathways, including those mediated by Ras and the mitogen‐activated protein kinases (MAPK)/extracellular signal‐regulated kinases (Erk), phospholipase Cγ (PLCγ), PI3K/Akt, NF‐κB, RhoA, and JNK cascades (Huang and Reichardt 2001; Roux and Barker 2002).

The mature neurotrophins are cleaved from proneurotrophins by intracellular furin, extracellular tPA (tissue plasminogen activator) or matrix metalloproteinases (MMP) (Arancio and Chao 2007). Proneurotrophins exert opposite effects to the mature ones through binding to different receptor complexes. Proneurotrophins favor interacting with p75^NTR^/sortilin more potently than mature neurotrophins and mediate apoptotic signals, while mature neurotrophins selectively bind to Trk receptors and mediate neuronal survival and regeneration. Studies showed that BDNF can promote learning and memory through activating TrkB, facilitating LTP in the hippocampus (Pang et al. 2004). In contrast, proBDNF interacts preferentially with p75^NTR^ together with the trafficking protein sortilin and can induce LTD and marked reduction in synaptic transmission in hippocampal slices. The effect of proBDNF likely involves enhancement of the expression of the NR2B subunit of the NMDA receptor (Woo et al. 2005; Rösch et al. 2005). Similarly, proNGF induces neuron death through binding with high affinity to the p75^NTR^‐sortilin receptor complex, which subsequently activates a small GTPase, Rac, and the proapoptotic JNK signaling cascade (Nykjaer et al. 2004; Harrington et al. 2002; Wang et al. 2010). Conversely, NGF selectively binds to p75^NTR^/TrkA and activates Ras/Erk1/2 (Susen et al. 1999) and NF‐κB‐mediated transcriptional mechanisms, inducing cell survival signaling (Carter et al. 1996). However, in the absence of TrkA, NGF/p75^NTR^ mostly mediates apoptotic pathways. p75^NTR^ signaling recruits various adaptor proteins such as NADE (neurotrophin receptor‐associated cell death executor), NRIF (neurotrophin receptor‐interacting factor), and NRAGE (neurotrophin receptor‐interacting MAGE homolog) that are associated with apoptosis, or RIP2 (receptor‐interacting protein 2), IL‐1 receptor‐associated kinase (IRAK), and TRAF‐6 (tumor necrosis factor receptor‐associated factor‐6) that promote survival; therefore, providing a bifunctional switch for cell survival or death (Mamidipudi et al. 2002; Khursigara et al. 2001, 1999).

p75^NTR^ may also associate with Nogo‐66 receptor (NgR) and LINGO‐1 (leucine‐rich repeat and immunoglobulin domain‐containing 1), and this receptor complex, when activated by myelin proteins such as myelin‐associated glycoprotein (MAG), oligodendrocyte myelin glycoprotein (OMgP), and Nogo‐66, inhibits axonal growth and prevents regeneration of injured nerve fibers through activation of the small GTPase RhoA (Yamashita et al. 1999; Charalampopoulos et al. 2012).

p75^NTR^ can interact with all three types of Trk receptors and modulate Trks' ligand‐binding affinity, presumably by conformational change (Siegel and Chauhan 2000). The binding of p75^NTR^ with TrkA promotes its autophosphorylation and enhances its binding affinity for NGF (Verdi et al. 1994), supporting the NGF/TrkA neuron survival through PI3K/Akt, NF‐κB pathways, and antiapoptotic Bcl‐2 expression (Culmsee et al. 2002). However, p75^NTR^ may have an opposite or negative effect on TrkB and C by reducing the autophosphorylation of TrkB in response to BDNF or NT‐4 (Vesa et al. 2000). Activation of the p75^NTR^‐Trk receptor complex with mature neurotrophins promotes cell survival and antagonizes the proapoptotic signaling cascade mediated by the p75^NTR^‐sortilin complex and proneurotrophins (Bibel et al. 1999). p75^NTR^ may also promote retrograde transport of neurotrophins (Curtis et al. 1995). Therefore, p75^NTR^ is considered a main regulator of cell survival and differentiation and can mediate either survival or degeneration depending on the ligand and pathway activated (Casaccia‐Bonnefil et al. 1999).

p75^NTR^ is primarily cleaved in endosomal membranes by TNF‐α‐secretase cleaving enzyme (TACE, also known as ADAM17) generating the soluble extracellular domain (p75ECD), which is the ligand‐binding site, followed by γ‐secretase to release its intracellular domain (p75ICD) (Zampieri et al. 2005). The metalloprotease‐mediated shedding of p75^NTR^ can be regulated by TrkA/NGF signaling and TrkB (Kanning et al. 2003; Urra et al. 2007). The p75ICD consists of a chopper domain and TNF‐like death domain and is thought to mediate neural apoptotic signals (Roux and Barker 2002; Majdan et al. 1997; Coulson et al. 2000).

#### 3.3.2 Neurotrophins in AD

Post‐mortem studies in AD revealed a severe reduction in the expression levels of BDNF (Phillips et al. 1991) and TrkA and B, upregulation of proNGF (due to decrease of proteolytic conversion) and sortilin (Fahnestock et al. 2001), and failure of retrograde transport of NGF signal in cholinergic neurons (Siegel and Chauhan 2000; Mufson et al. 1995). Interruption of NGF and BDNF signaling activates amyloidogenesis and favors apoptotic cell death via proNGF/p75^NTR^ signaling in hippocampal neurons (Matrone et al. 2008; Pedraza et al. 2005). NGF controls both APP phosphorylation at T668 as well as subcellular trafficking and localization of APP and BACE1. NGF inhibits JNK‐mediated phosphorylation of APP at T668, which in turn favors TrkA interaction with APP. This interaction favors α‐secretase cleavage of APP and induces its trafficking to the Golgi where APP‐BACE interaction and cleavage is inhibited (Triaca et al. 2016). The potential significance of NGF in amyloidosis arises from the fact that TrkA fails to interact with phosphorylated APP T668. In AD, these events are severely disrupted where pAPP T668 highly accumulates in dystrophic neurites and plaques, and APP‐TrkA interaction is inhibited. In a positive feedback loop, Aβ may aggravate and participate in neurotoxicity induced by neurotrophin imbalance. Aβ could inhibit TrkA phosphorylation and ubiquitination and disrupt its association with p75^NTR^ and NGF; hence, it inhibits TrkA neuron survival signaling pathways (Zheng et al. 2015). Tiernan et al. provided evidence for a link between pre‐tangle pathology and neurotrophin imbalance within the cholinergic nucleus basalis neurons. Disrupted neurotrophin signaling contributes to and coincides with the progression of NFT pathology in AD patients (Tiernan et al. 2018), and in turn, phosphorylated tau (pS422+ neurons) may impair NGF retrograde transport (Tiernan et al. 2016) and inhibit TrkA expression and therefore support the shift from pro‐survival to proapoptotic signaling (Tiernan et al. 2018).

#### 3.3.3 p75^NTR^ in AD and FTD

p75^NTR^ pan receptor is a member of the TNF superfamily that is mainly expressed in the basal forebrain cholinergic neurons, the dorsal root ganglion, and sympathetic neurons. It is upregulated in aging and in response to stress conditions such as OS, inflammation, and brain injury, altering the ratio of p75^NTR^ to Trk in favor of p75^NTR^ (Ibáñez and Simi 2012). p75^NTR^ has diverse roles and activates three main pathways, including neuronal growth, apoptosis, and synaptic plasticity (Zeng et al. 2011). It is also involved in neuronal cell cycle re‐entry, which is believed to be a key pathological mechanism underlying the development of AD and other neurodegenerative diseases (Frade and López‐Sánchez 2010).

Several studies proved that p75^NTR^ mediates Aβ‐induced neurotoxicity (Knowles et al. 2009; Sotthibundhu et al. 2008) and tau hyperphosphorylation (Shen et al. 2018) and regulates Aβ deposition and metabolism in the brain (Wang et al. 2011). Similar to proneurotrophins, Aβ (both soluble oligomeric and aggregated) can bind to p75^NTR^, inducing apoptotic signals and neurite collapse through upregulation of JNK expression and phosphorylation (Yaar et al. 1997) and caspases, as well as through the RhoA/ROCK pathway, triggering spine loss (Patnaik et al. 2020). However, low concentrations of Aβ (in the nanomolar to picomolar range) can mediate p75^NTR^‐induced neurite growth, like NGF, through induction of the Ras–Erk pathway (Susen and Blöchl 2005). Aβ can upregulate p75^NTR^ (Chakravarthy et al. 2010) and sortilin expression through p75^NTR^/RhoA signaling pathways (Saadipour et al. 2013). Activation of p75^NTR^ and sortilin pathways enhances BACE1 expression and trafficking (Finan et al. 2011) and induces APP phosphorylation and localization into endosomes through JNK activation, thereby maintaining a vicious cycle of amyloidosis and neurodegeneration (Saadipour et al. 2018, 2019). Matrone et al. showed that accumulation of Aβ in AD, due to failure of NGF signal, may induce TrkA phosphorylation and interaction with p75^NTR^ and PS1, shifting the neurotrophic signal of TrkA to the apoptotic signal of p75^NTR^ via the activation of phospholipase Cγ (PLCγ) and catalyzed by Src and CDK5 kinases (Matrone et al. 2009). p75^NTR^ also plays a major role in Aβ uptake and clearance through inducing lysosomal internalization and degradation of the Aβ‐p75^NTR^ complex in the basal forebrain cholinergic neurons (Ovsepian et al. 2014); however, this process may be impaired in degenerated neurites. Wang et al. showed that deletion of p75^NTR^ in APP/PS1 AD mice reduced soluble Aβ levels in the brain and serum but increased plaque accumulation (Wang et al. 2011). Delivery of p75ECD‐Fc recombinant protein into the hippocampus of AD mice suppressed plaque aggregation, attenuated synthetic Aβ_42_ peptide oligomerization and fibrillation, and solubilized fibrilized Aβ in vitro (Wang et al. 2011; Yao et al. 2015). These findings indicated that p75^NTR^ plays a critical role in Aβ production, possibly via the NGF/p75^NTR^ signaling pathway (Costantini et al. 2006), and highlighted the possible role of the p75 extracellular domain in sequestering Aβ, reducing its aggregation, and promoting its clearance similarly to that of anti‐Aβ antibodies. Therefore, p75^NTR^ plays two opposing roles in Aβ homeostasis in the brain. On one side, p75^NTR^ signaling may increase Aβ production and maintain steady‐state levels of Aβ. On the other side, p75ECD, after shedding from the membrane, may bind and sequester Aβ, thus suppressing its aggregation in the brain and enhancing its clearance across the BBB. Moreover, the brains of FTD‐tau patients showed elevated expression levels of p75^NTR^ and proNGF, while knockdown of p75^NTR^ in P301L tau transgenic mice attenuated the proNGF‐induced tau hyperphosphorylation via activation of the Akt/GSK‐3β pathway (Shen et al. 2018; Mañucat‐Tan et al. 2019).

Yao et al. found that the levels of p75ECD are reduced dramatically in the brain and CSF (cerebrospinal fluid) in AD, while the levels of p75 full‐length increased, which is possibly due to the Aβ‐induced reduction in the expression and activity of the cleaving enzyme TACE (Yao et al. 2015). Petri et al. suggested that Aβ dose‐dependently inhibits TACE through the activation of the PDK1 (3‐phosphoinositide‐dependent kinase‐1) signaling pathway, which triggers TACE internalization and reduces the cell‐surface activity of TACE (Pietri et al. 2013). TACE inhibition reduces the shedding of the p75 extracellular domain and accumulates the full‐length receptor (p75 fl), leading to aggravation of the apoptotic p75^NTR^ signaling pathway mediated by Aβ and proneurotrophins. Consequently, restoration of p75ECD levels could sequester Aβ, inhibit its aggregation, and may serve as a competitive antagonist on p75^NTR^ full‐length by antagonizing the binding of Aβ with p75^NTR^. Treatment of AD transgenic mice with AAVECD‐Fc administered either in one of the lateral ventricles or peripherally improved learning and memory and reduced AD pathologies, including amyloid plaques, tau phosphorylation, cell death, synaptic degeneration, and neuroinflammation (Yao et al. 2015; Wang et al. 2016). Similarly, AAVECD‐Fc alleviated tau pathology and neurodegeneration in P301L mice (Shen et al. 2018, 2019). p75ECD also blocked Aβ‐ and proBDNF‐induced tau phosphorylation and neurotoxicity in cultured neurons (Mañucat‐Tan et al. 2019). Therefore, p75ECD is considered a potential diagnostic biomarker and novel therapeutic approach for the treatment of AD and tauopathies (Zhou and Wang 2011).

Conversely, the increased levels of p75ECD in the blood of AD patients could differentiate AD from cognitively normal subjects (Jiao, Bu et al. 2015). The source of p75ECD in the blood is still elusive since p75 mRNA levels in peripheral blood cells are not altered between the two groups (Xu et al. 2019).

### 3.4 Mitochondrial Dysfunction and OS Hypothesis

OS has been identified as a contributing factor in aging and in the progression of several neurodegenerative diseases, including AD (Tönnies and Trushina 2017), FTD‐tau (Martínez et al. 2008; Gerst et al. 1999) and amyotrophic lateral sclerosis (Ilieva et al. 2007). Evidence from studies on iPSC‐derived early sporadic AD patient neuronal cells showed increased production of ROS and dysfunction of the mitochondrial respiratory chain prior to Aβ and tau accumulation (Birnbaum et al. 2018). Oxidative damage has also been found to underlie different subtypes of FTD but more prominently in FTD‐tau cases (Martínez et al. 2008). Kamat et al. in 2014 suggested that “oxidative stress mediated through NMDAR and their interaction with other molecules might be a driving force for tau hyperphosphorylation and synapse dysfunction” (Kamat et al. 2016).

Mitochondria are considered the main source of intracellular oxidant production (Holmström and Finkel 2014). Accordingly, Swerdlow et al. suggested the mitochondrial cascade hypothesis, which states that “in sporadic, late‐onset AD, loss of mitochondrial function associated with age affects the expression and processing of APP, initiating Aβ accumulation” (Swerdlow et al. 2014; Swerdlow and Khan 2009).

**FIGURE 5:**
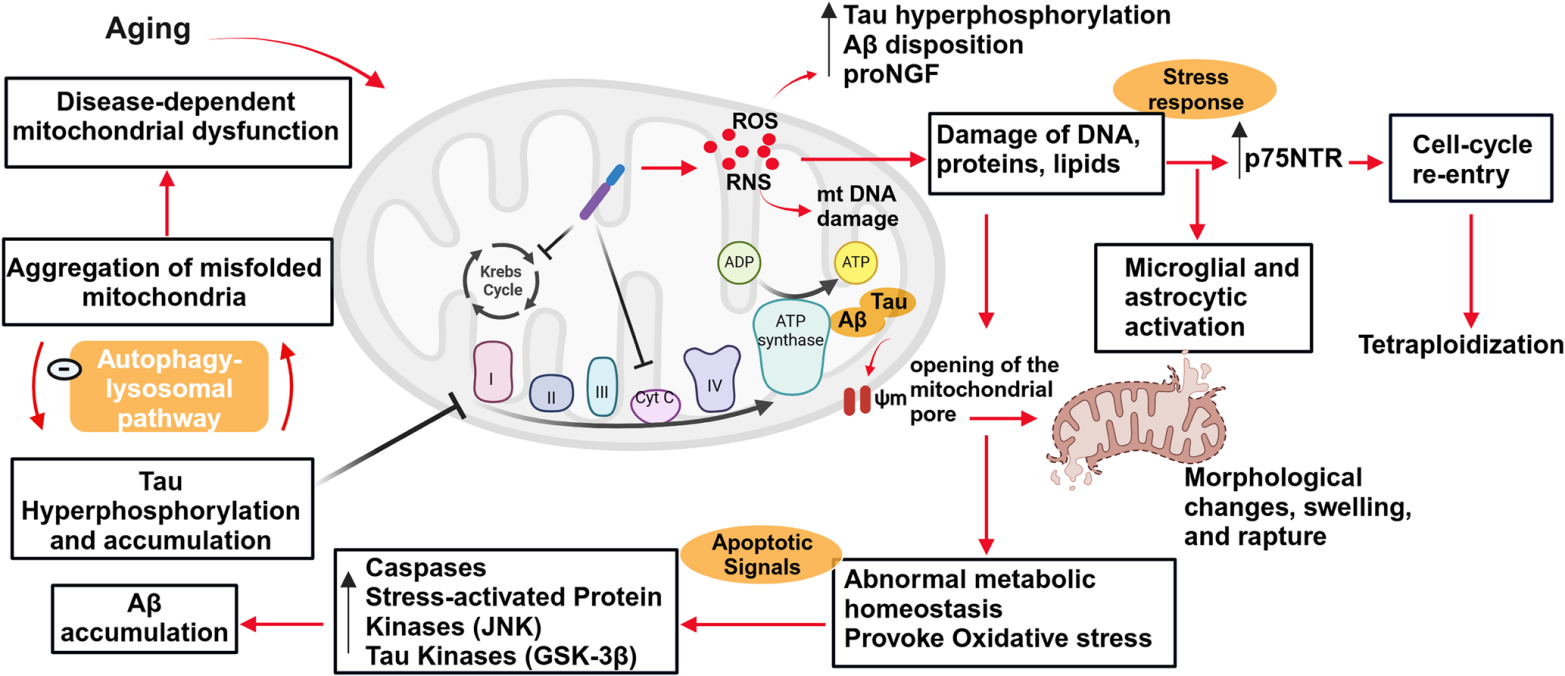
Mitochondrial dysfunction and Oxidative stress in AD. Cellular Aβ attacks cytochrome C (complex IV) in the electron transport chain and Krebs‐cycle enzymes and induces the production of reactive oxygen and nitrogen species (ROS, RNS). Increased oxidative stress damages the mitochondrial DNA and nuclear DNA, lipids, and proteins, leading to stimulation of the stress response mediated by proNGF and p75NTR. This, in turn, provokes abnormal Aβ production and disposition and tau hyperphosphorylation. Upregulation of p75NTR receptors induces an inflammatory response through activation of microglia and astrocytes and forces towards cell cycle re‐entry and formation of tetraploids. Aβ and pathogenic tau induce the loss of mitochondrial membrane potential and opening of the permeability‐transition pores (ψm). This results in mitochondria swelling and rupture that further exacerbates OS, apoptotic signaling through Caspase‐3 and stress‐activated protein kinases p53 and c‐jun‐N‐terminal kinase; JNK, in addition to tau kinases (GSK‐3β). The activation of these pathways provokes abnormal Aβ accumulation and tau hyperphosphorylation that, in turn, interferes with complex I in the electron transport chain. Misfolded pathogenic tau also inhibits the clearance of misfolded proteins and degradation of impaired mitochondria through interfering with the autophagy‐lysosomal pathway (ALP). Conversely, mitochondrial dysfunction aggravates ALP impairment and contributes to tau accumulation. This cycle further exacerbates disease‐dependent mitochondrial dysfunction induced by aging and disrupted antioxidant defenses.

Increased production of reactive oxygen species (ROS) and reactive nitrogen species (RNS) due to aging and disease‐dependent mitochondrial dysfunction as well as insufficient antioxidant defenses induce synaptic damage and neurotoxicity. Oxidative damage to cellular components, including nuclear and mitochondrial DNA, lipids, and proteins likely results in abnormal metabolism and metal ion homeostasis, activation of caspases and stress‐activated protein kinases, and main tau kinases, such as GSK‐3β, inhibition of phosphatases, and inflammation (Feng et al. 2013). These events could represent the initial trigger that could enhance the production and accumulation of Aβ and hyperphosphorylated tau. In a positive feedback loop, Aβ and tau could independently exacerbate mitochondrial dysfunction and OS, contributing to a vicious cycle that exacerbates with time, leading to neuronal death (Tönnies and Trushina 2017; Mattson and Magnus 2006; Yao et al. 2009) (Figure 5). Tau oligomers may interfere with the autophagy‐lysosomal pathway (ALP), which plays a role in the clearance of misfolded proteins (Wang et al. 2009; Lim et al. 2001) and degradation of impaired mitochondria. Conversely, mitochondrial dysfunction aggravates ALP impairment and contributes to tau protein accumulation in AD and FTD‐tau (Lim et al. 2001; Silva et al. 2011). According to Fade et al. “aging‐dependent oxidative stress represents a crucial initiator of neurodegeneration, causing covalent modification of different molecules, enhancing the expression of proNGF and other key regulators of the cell cycle as well as of p75^NTR^ that forces cell cycle re‐entry and neuronal tetraploidization and eventually leads to microglial and/or astrocytic activation and setting the cell to stress response” (Frade and López‐Sánchez 2010). Polyploid cells have been detected in the frontal cortex of both control and AD brains but to a greater extent in AD brains (Iourov et al. 2009; Mosch et al. 2007).

### 3.5 The Inflammation Hypothesis

Krstic et al. suggested that inflammation represents a major trigger to the neuropathology of sporadic AD, referred to as “the inflammation hypothesis of AD” (Krstic and Knuesel 2013). Chronic inflammation and cellular stress to neurons during aging caused by disease (i.e., diabetes), infection, or age‐related changes induce tau phosphorylation and misfolding that result in impaired axonal transport. Such changes might hinder protein extrusion mechanisms and induce focal axonal swellings and blockage with concomitant accumulation of mitochondria, leading to disturbed energy metabolism and ultimately axonal leakage and loss of synaptic connections. This state of chronic inflammation and defective axonal transport promotes APP accumulation and amyloidogenic processing, which subsequently activates the innate immune system and induces priming and recruitment of microglia and astrocytes to the degenerating neurons. Microglia and astrocytes become over‐activated and release inflammatory mediators that will eventually extend to the nearby neurons. Chronic inflammatory activation of microglia releases adaptor protein apoptosis‐associated speck‐like protein containing a CARD (ASC) that assembles into ASC specks, which can sustain an ongoing immune response and directly induce Aβ seeding and spreading of the pathology (Venegas et al. 2017; Bettcher et al. 2021). This hypothesis is supported by the observations of Braak and colleagues, who recently reported that tau‐related neuronal changes appeared before amyloid deposits and that in more than half of the investigated cases, abnormal tau protein occurred without the presence of Aβ deposits (Braak et al. 2011). These findings indicate that the formation of hyperphosphorylated tau induced by inflammatory states which lead to impairments in neuronal integrity, could represent an early neuropathological event that precedes deposition of the classical AD hallmarks (Krstic and Knuesel 2013).

In the same context, growing evidence indicates that dysfunction of both the peripheral and central immune system responses is an early event of dementia, including AD, and suggests the role of innate and adaptive immune responses in accelerating neurodegeneration (Bettcher et al. 2021; Qian et al. 2022; Lai et al. 2022). In AD patients, increased infiltration of peripheral B lymphocytes and subclasses of T cells (CD8+ T cells) in the brain parenchyma was observed, as well as alteration of natural killer function, which could initiate neuroimmune responses, induce the glial proinflammatory response, and contribute to cytotoxicity and neurodegeneration in dementia (Kim et al. 2021; Solerte et al. 2000; Merlini et al. 2018). Although results from animal studies suggest a beneficial role of regulatory T cells in slowing disease progression in early stages through recruiting microglia towards Aβ deposits (Baruch et al. 2015; Dansokho et al. 2016). In line with these observations, Lai et al. (2022) discovered five distinct immune‐related genes—CXCR4, PPP3R1, HSP90AB1, CXCL10, and S100A12—that show altered expression in AD. Findings from epidemiological studies further showed that elevated levels of baseline peripheral inflammatory markers in healthy adults, such as C‐reactive protein, TNF‐α and IL‐1β, are associated with a high risk of future development of all‐cause dementia, including AD (Tan et al. 2007; Engelhart et al. 2004). Also, a chronic inflammatory state is linked to earlier onset of the disease in *APOEε4* carriers (Tao et al. 2018). The Atherosclerosis Risk in Communities (ARIC) study indicated that a high extent of systemic inflammation during midlife is a driver of fast and steeper cognitive decline (Walker et al. 2019). Alteration of the peripheral immune response and early proinflammatory cytokine changes were also observed in patients with FTD spectrum and vascular dementia, but each disorder shows unique changes to those found in AD, indicating different underlying disease mechanisms (Busse et al. 2017; Katisko et al. 2020). Thus, strategies targeting the immune system to modulate neuroinflammation could be effective therapeutic approaches for the prevention of AD and treatment of other neurodegenerative disorders (Bettcher et al. 2021; Haage and De Jager 2022).

### 3.6 Insulin Resistance and Neuro‐Metabolic Hypothesis

The concept of brain insulin dysfunction in AD was initially developed by Siegfried Hoyer in 1985–2000 (Morgen and Frölich 2015; Hoyer 2002). Research has found that disturbances in brain insulin signaling could result in a cascade of damaging events such as failure of oxidative energy metabolism, dysfunction of various cellular organelles and cell cycle, and impairment of cerebral blood flow; these metabolic abnormalities can aggravate the accumulation of toxic Aβ and hyperphosphorylated tau proteins. Thus, dysfunctional brain insulin signaling may be a contributing factor to the late‐onset AD (Morgen and Frölich 2015; Polis and Samson 2019). Clinical studies showed that the type 2 diabetic patients are at an increased risk of developing dementia during aging (Barbagallo and Dominguez 2014). Factors related to metabolic syndrome, such as hyperlipidaemia, obesity, and hypertension are linked to diabetes and may contribute to dementia (Biessels and Kappelle 2005; Kang et al. 2017). Also, patients with advanced AD may present with high plasma insulin levels, increased lactate levels in the CSF, low CSF insulin levels, and a reduced CSF/plasma insulin ratio compared to healthy age‐matched adults (Craft et al. 1998), reflecting a progressive peripheral insulin resistance. Imaging tools have also revealed early selective atrophy and altered glucose metabolism in the medial temporal lobe of AD patients (Jeong et al. 2016). In addition, an impaired insulin transduction mechanism and loss of tyrosine kinase activity were reported in AD brains with a compensatory increase in the number of insulin receptors (IRs) compared to the age‐matched controls. (Frölich et al. 1999). In addition, the expression levels of several components of the insulin signaling pathway were found to be altered in the brains of AD patients. In this regard, levels of insulin‐like growth factor IGF (I and II), their corresponding receptors and mRNA levels of insulin receptor substrate (IRS) were reduced. This was associated with the reduced levels of phosphatidylinositol 3‐kinase (PI3K) and phospho‐Akt/Protein Kinase B (the active form), but increased activity of the GSK‐3β kinase (reduced phosphorylated GSK‐3β levels) as well as the increased APP mRNA expression (Steen et al. 2005). These changes were also observed in age‐matched healthy controls (age > 60), suggesting that aberrant insulin signaling is age‐related and can precede or accompany the initial stages of cognitive decline (Hoyer 2004). Brain insulin resistance reduces neuronal energy/glucose metabolism and will eventually induce defects in synaptic integrity and render the neurons susceptible to other cellular insults such as inflammation, OS, and mitochondrial dysfunction. Defects in the activity of the pyruvate dehydrogenase complex and ketoglutarate dehydrogenase complex in the brain, which are the key energy‐related enzyme complexes in mitochondria, have been detected in post‐mortem AD brain and are a possible consequence of the aberrant insulin signaling (Blass et al. 2002). Zhao et al. showed that Aβ oligomers induced a rapid and substantial loss of neuronal surface IRs in hippocampal neurons in vitro (specifically on dendrites) and inhibited insulin receptor signaling (Zhao et al. 2008). Aβ oligomers caused a persistent increase in Akt phosphorylation at Ser473, which is a main component in the negative feedback loop on PI3K and IRs that contributes to insulin resistance in AD brain (Pessin and Saltiel 2000). Therefore, the term “type 3 diabetes” has been proposed to describe the pathophysiology of insulin resistance brain state of sporadic AD (Steen et al. 2005; de la Monte and Wands 2008).

## 4 Current and Novel Therapeutic Strategies

### 4.1 Currently Approved Therapies for AD and FTDri

Current symptomatic treatments for AD include acetylcholinesterase inhibitors (donepezil, galantamine, rivastigmine, and tacrine) and a low‐affinity NMDA receptor antagonist (memantine), for moderate to severe AD. The acetylcholinesterase inhibitors mediate their effects by partially alleviating the cholinergic deficit in AD (Deardorff et al. 2015), whereas the precise mechanism of action of memantine remains elusive, but it is thought to interfere with the neurotoxic effects of Aβ mediated on the NMDA receptor (Rogawski and Wenk 2003). Generally, the effects of these drugs are quite limited, as they somewhat improve some of the symptoms but do not treat the underlying causes of the disease (Karran et al. 2011).

To date, only one disease‐modifying treatment approved in the USA for FTD is Riluzole. Current symptomatic treatments include antidepressants such as selective serotonin reuptake inhibitors and trazodone for disinhibition, depression, and compulsive behavior, and second‐generation antipsychotics such as olanzapine, quetiapine, and aripiprazole to treat aggression, agitation, and psychosis. Unfortunately, so far, no treatment proven to be safe and effective is available to alleviate motor symptoms that associate FTD with parkinsonism (FTDP‐17) (Panza et al. 2020). Non‐pharmacological interventions, including exercise, physical and speech therapy, as well as introducing behavioral management approaches and environmental modifications were found to help alleviate behavioral symptoms, stabilize speech and language, and reduce abnormal motor behavior of FTD (Magrath Guimet et al. 2022).

### 4.2 Novel Therapeutic Strategies

#### 4.2.1 Therapies Targeting Aβ Production, Accumulation, and Clearance

So far, nearly all Aβ‐targeted phase III clinical trials in AD have not shown statistically significant benefits on their clinical endpoints, and they were discontinued due to side effects that are most likely not target‐related (De Strooper and Karran 2016; Selkoe and Hardy 2016; Karran et al. 2011).

##### 4.2.1.1 Modulation of β‐ and γ‐Secretase Activity

Clinical trials are carried out to investigate the safety and efficacy of selective BACE1 inhibitors. However, the clinical development of almost all these compounds, such as Verubecestat, Atabecestat, Umibecestat, and Elenbecestat, has been terminated due to severe adverse events and lack of significant improvement in cognition scores (Madav et al. 2019).

Similarly, the clinical development of various γ‐secretase inhibitors such as Semagacestat and Avagacestat was discontinued as they were neither effective nor safe due to severe adverse events that might be caused by a lack of selectivity to APP and Notch (Doody et al. 2013; Coric et al. 2012).

##### 4.2.1.2 Aβ Vaccination and Anti‐Aβ Antibodies (Immunization Therapies)

Active Aβ immunization, such as (AN1792) (Gilman et al. 2005) and LuAF20513 (Davtyan et al. 2013), and anti‐Aβ antibodies (Bapineuzumab, Solanezumab, Aducanumab, Gantenerumab and Ponezumab) (Plotkin and Cashman 2020) were developed as a potential therapeutic approach for reducing levels of brain Aβ (Table 1). Antibodies were designed to target the mid‐region of Aβ and bind primarily to monomers and low‐n oligomers. These antibodies cross the BBB and sequester Aβ in the brain and enhance the phagocytosis of the Aβ/antibody complex by microglia through opsonization (Schenk et al. 1999; Bard et al. 2000). They may also act by capturing Aβ in the periphery and inhibiting its uptake to the brain, which will consequently accelerate Aβ efflux from the brain and promote deposited plaque dissociation (DeMattos et al. 2002, 2001). Both active and passive vaccination were successful in AD animal models. However, the clinical development of most Aβ immunotherapies was halted due to severe immune responses such as meningoencephalitis, microhemorrhage, and vasogenic edema. These adverse effects might be due to the direct interaction of antibodies with Aβ in the brain that might hinder the efflux of this complex to the blood and become trapped in the brain vascular wall, weakening the blood vessels in the brain and inducing inflammatory responses and bleeding and resulting in the accumulation of multimeric toxic Aβ species in the brain (Yamada et al. 2009). Also, autoimmune meningoencephalitis might be caused by the abnormal infiltration of T lymphocytes into the CNS activated by the T cell‐activating domain of Aβ vaccine (Liu et al. 2014). In this respect, the FDA granted accelerated approval in June 2021 to Aducanumab, a fully human IgG1 monoclonal antibody against Aβ epitope 3–7, based on its effect on a surrogate endpoint that likely predicts its clinical benefits, but post‐approval trials were conducted to verify its expected clinical benefits (Budd Haeberlein et al. 2022). Aducanumab binds to soluble Aβ aggregates and insoluble fibrils with much higher selectivity over monomers and can also inhibit secondary nucleation (Morató et al. 2022). Unfortunately, in April 2022, the manufacturing company withdrew its application for marketing authorization of Aduhelm (Aducanumab) for the treatment of AD, based on the European Medicines Agency's recommendation to refuse marketing authorization in December 2021. Clinical trials found that Aducanumab reduced Aβ in the brain; however, the link between this effect and clinical improvement had not been established. Also, the results were conflicting and did not convincingly show that Aducanumab was effective at treating early‐stage AD patients. Besides, images from brain scans of some patients showed amyloid‐related imaging abnormalities (ARIA)—edema suggesting the presence of swelling or bleeding that could be potentially harmful. In January 2023, the FDA approved Lecanemab (Leqembi), another monoclonal antibody developed by Eisai and Biogen, as it has been shown to slow cognitive decline in a robust Phase II clinical trial and decrease amyloid and tau accumulation in patients' brains, with much less incidence of brain swelling or bleeding compared to Aducanumab (van Dyck et al. 2022; Larkin 2023; Reardon 2023). In July 2023, Leqembi received FDA traditional approval for the treatment of early AD patients and those with MCI (Harris 2023). Donanemab is another monoclonal antibody developed by Lilly that significantly slowed cognitive and functional decline in Phase II clinical trials in patients with early AD (Mintun et al. 2021). The U.S. FDA has recently approved it under the name of Kisunla for patients with MCI or mild stages of the disease (Wicker et al. 2024). However, the biggest concern is the adverse effects that can lead to bleeding and seizures in patients receiving Donanemab compared to placebo, and that would be pronounced especially in those who are taking anticoagulants (Shcherbinin et al. 2022; Rashad et al. 2022). Emerging monoclonal antibodies that are currently under development include Trontinemab, Remternetug (LY3372993), PRX012, and ACU193. Some have progressed to phase III clinical trials and show promising preliminary results with more efficient and quick removal of Aβ (Wicker et al. 2024; Grimm et al. 2023; Tam et al. 2021; Siemers et al. 2023).

In order to minimize Aβ vaccine‐induced T‐cell autoimmunity, second‐generation Aβ targeting vaccines were designed using different platforms such as ACC‐001 (Aβ 1–7 peptide conjugated to diphtheria toxoid protein), CAD106 (Aβ 1–6 peptide coupled to bacteriophage Qb carrier), V950 (multivalent Aβ 1–15), AFFITOPES AD01 and AD02 (Aβ mimetics conjugated to KLH carrier; Keyhole Limpet Hemocyanin) and ACI‐24 (liposome vaccine‐based array of Aβ1–15 sequences). Some of these new vaccines progressed to phase 3 trials but, unfortunately, no differences were observed between treatment and placebo groups regarding brain volume and cognitive decline, possibly because they could not elicit a significant and sustained anti‐Aβ‐antibody response. UB‐311 is another novel active Aβ synthetic vaccine constructed with two synthetic N‐terminus Aβ1–14–targeting peptides (B‐cell epitope), each coupled to different helper T‐cell peptide epitopes (UBITh‐platform‐based vaccine) and developed on a Th2‐biased delivery system. UB‐311 has proven to be safe and well‐tolerated and generated a significant anti‐Aβ antibody response against both Aβ1–42 monomers and toxic oligomers (Wang, Wang, Chiu, et al. 2017) compared to other active vaccines. In addition, patients with mild AD showed cognitive improvement in phase 2 clinical trials, though phase 3 trials have not been launched yet (Morató et al. 2022). ABvac40 is also an active anti‐Aβ vaccine, but it is targeted against the C‐terminal end of Aβ40 and coupled to KLH carrier. ABvac40 showed a favorable safety and tolerability profile since no cases of vasogenic edema or microhemorrhages have been recorded among participants in phase I trials (Lacosta et al. 2018). Phase II clinical trials are ongoing to confirm its safety and tolerability and to investigate its clinical efficacy in patients with MCI and very mild AD. AOE1 is a yeast cell‐based vaccine recently developed by Yu et al. and Wang et al. that targets a specific conformational epitope of Aβ42 oligomers (Yu, Zhu, et al. 2020). AOE1 effectively attenuated neuropathology and cognitive deficits in APP/PS1 mice and did not induce T cell activation or microhemorrhages, suggesting that it may be a safer and more effective vaccine for AD treatment (Wang, Liu, et al. 2017).

##### 4.2.1.3 Small‐Molecule Aβ Anti‐Oligomer and Aggregation Inhibitor

Recently, ALZ‐801 (Valiltramiprosate) was designed as an oral valine‐conjugated prodrug of Tramiprosate, which is a small molecule inhibitor of amyloid oligomer formation and aggregation through stabilizing Aβ42 monomers (Hey et al. 2018). Preclinical data and Phase I trials showed that ALZ‐801 has improved pharmacokinetic (PK) properties and has a high brain penetration. Its efficacy and tolerability are currently being evaluated in early AD patients who are carriers of *APOEε4/4* or *APOEε3/4* genotypes.

#### 4.2.2 AD Therapies Based on Neurotrophins

##### 4.2.2.1 NGF Gene Delivery

According to the neurotrophin hypothesis for AD, therapies aimed at restoring the homeostatic balance between ligands and receptors of the neurotrophin pathway appear to have a truly disease‐modifying efficacy (Cattaneo and Calissano 2012). However, therapeutic application of neurotrophins has been limited so far owing to their poor plasma stability (half‐life of a few minutes or less), oral bioavailability, and restricted ability to penetrate the BBB. Besides, their pleiotropic actions, triggered by acting on a complex receptor signaling network, could lead to adverse effects such as hyperalgesia with NGF treatment (Longo and Massa 2013). For example, stereotaxic gene delivery of NGF (CERE‐110) proved to be effective and well‐tolerated in a phase I clinical trial (Rafii et al. 2014) (ClinicalTrials.gov Identifier: NCT00087789). But the invasiveness of the procedure and adverse events encountered by participants, such as severe back pain and weight loss, have limited its clinical development (Rafii et al. 2014; Eriksdotter Jönhagen et al. 1998; Tuszynski et al. 2005).

##### 4.2.2.2 Small Molecule Neurotrophin Ligands

A recent approach developed by Longo et al. involves the use of small molecule ligands that “may bind to receptors at sites that would otherwise be occupied by only one of the several domains within a bound protein ligand” (Longo and Massa 2013). Such binding may or may not result in substantial displacement of the native ligand from the receptor and may promote conformational changes that activate the receptor. These ligands focused on the modeling of binding domains within NGF, BDNF, or NT3. Examples of small molecule ligands are 7,8 dihydroxyflavone, a TrkB binding small molecule; and LM11A‐24 and LM11A‐31, which are neurotrophic p75^NTR^‐binding non‐peptide small molecules that structurally mimic loop1 of NGF. LM11A‐24 and LM11A‐31 displayed good oral bioavailability and penetration of BBB in vivo (Tep et al. 2013), inhibited proNGF induced apoptosis in oligodendrocytes and Aβ‐induced toxicity in hippocampal neurons (Massa et al. 2006), and improved recovery after spinal cord injury in animals (Tep et al. 2013). Surprisingly, LM11A‐24 and LM11A also alleviated behavioral deficits, reversed neurite dystrophy, and reduced tau misfolding and phosphorylation as well as inflammatory changes in the hAPP^Lond/Swe^ and Tg2576 mouse models of AD (Knowles et al. 2013; Simmons et al. 2014; Nguyen et al. 2014). Another study showed that oral administration of LM11A‐31 led to a significant improvement of erectile function induced by cavernous nerve injury in C57BL/6 mice (Yin et al. 2021). These findings support the possible clinical benefits of LM11A‐31 in the treatment of erectile dysfunction in men after radical prostatectomy or with neurovascular disorders. Another recent study found that poststroke oral administration of LM11A‐31 in mice attenuated brain metabolic changes, increased neuronal survival and neurotransmitter levels and improved recovery, suggesting that LM11A‐31 could be a possible candidate in stroke (Nguyen et al. 2022). Currently, phase I/II clinical trials of LM11A‐31 in mild–moderate AD patients have been completed, but results have not been released yet (Table 1).

##### 4.2.2.3 p75ECD‐Fc

Recent studies also focused on restoring the balance between the neuroprotective p75ECD and p75^NTR^ full‐length receptor in the brain. Wang and Zhou's group has made a significant contribution to understanding the roles of p75^NTR^ and its extracellular domain in the pathogenesis of AD (Zhou and Wang 2011; Jiao, Bu et al. 2015). They showed that brain delivery of AAV‐ECD reduced cognitive deficits and neuropathology in AD mice (Yao et al. 2015). Furthermore, the intramuscular injection of AAV‐p75ECD reduced Aβ production and tau phosphorylation and improved cognitive deficits (Wang et al. 2016). p75ECD‐Fc is a human recombinant fusion protein of p75NTR extracellular domain‐human IgG Fc fusion protein (patency: US11046746B2), that was first synthesized in cooperation with Fujian Tiantai and Wuxi APPtec in China. Treatment with p75ECD‐Fc inhibited the proNGF/p75NTR‐induced tau phosphorylation in cultured neurons and improved memory functions and suppressed tau pathology in P301L, a mouse model of FTDP‐17 (Shen et al. 2018). Another study showed that intravenous injection of p75ECD‐Fc attenuates myocardial ischemia–reperfusion injury in rats by inhibiting myocardial proneurotrophin expression and blocking the JNK/caspase pathway (Fang et al. 2020). Jiao, Bu, et al. found that CSF levels of p75NTR‐ECD decreased while serum levels increased in AD patients. These changes are strongly correlated with Mini‐Mental State Examination scores, suggesting that p75NTR‐ECD levels could be a specific diagnostic marker for AD and monitoring AD progression (Jiao, Bu et al. 2015). In this context, a quantification study of p75NTR‐ECD levels in CSF of patients with AD, MCI due to AD, FTD, and non‐neurodegenerative dementia was completed, but the results have not been released yet (ClinicalTrials.gov Identifier: NCT02946710).

It is proposed that p75ECD‐Fc may act through two possible mechanisms: first, it acts centrally as a scavenger of Aβ and proneurotrophins and competitively antagonizes their neurotoxic effects by blocking the activation of the p75^NTR^ full‐length receptor. Second, p75ECD‐Fc may act like an anti‐Aβ antibody which binds Aβ forming Aβ‐p75ECD‐Fc complex that is recognized and phagocytosed by the brain microglia and resident macrophages in the periphery through its Fc tail. Thus, p75ECD‐Fc may capture Aβ and enhance its peripheral clearance, which will shift the balance towards Aβ efflux from the brain to the periphery and, therefore, promote plaque dissociation and clearance from the brain (Wang et al. 2016). This is referred to as the “peripheral sink mechanism” for peripheral Aβ clearance (Liu et al. 2014). Such approach is expected to have minimal side effects compared to those observed with Aβ antibodies due to its structural similarity with the endogenous molecule and will not result in central immune‐related adverse events, possibly due to enhancing the peripheral Aβ clearance. Preclinical studies showed that p75ECD‐Fc exhibits a long half‐life of ~7 days, following peripheral administration, with limited ability to cross the BBB and high distribution in the liver and kidney, where it was found colocalized in resident macrophages in these tissues (Kelliny et al. 2020). Colocalization of the drug in resident macrophages may support the hypothesis that p75ECD‐Fc could act through scavenging Aβ, promoting its clearance in the periphery and subsequently reducing the levels in the brain.

Clearance of Aβ through brain microglia is inefficient in AD due to defective phagocytic ability that is caused by persistent inflammatory responses. However, this process can be enhanced by Aβ‐binding molecules, such as anti‐Aβ antibodies (Lai and McLaurin 2012). Apart from enzymatic degradation of Aβ in the brain (via IDE, neprilysin and other enzymes) (Wang et al. 2006), Aβ is transported via the interstitial fluid (ISF) into the cerebrospinal fluid (CSF) via bulk flow, but this has been proved to be a minor route for Aβ clearance, as it only accounts for nearly 10% of Aβ clearance (Shibata et al. 2000). The other main route of Aβ clearance from the brain is believed to occur through direct trafficking out of the brain via LDL receptor–related protein‐1 (LRP‐1) and P‐glycoprotein (Shibata et al. 2000) at the BBB into peripheral circulation, through which 40%–60% of brain Aβ is cleared mainly through the liver and kidney (Karran et al. 2011). Wang and colleagues have provided clear evidence of the significance and efficacy of peripheral Aβ clearance in AD by constructing a parabiosis model in AD mice and found that peritoneal dialysis reduced brain Aβ burden and neuropathology in AD mice (Xiang et al. 2015). They also found that plasma Aβ levels significantly decreased immediately after peritoneal dialysis in patients with chronic kidney disease (Jin et al. 2017). In addition, levels of brain Aβ increased significantly in chronic kidney disease, or cirrhosis patients, indicating the substantial impact of peripheral organs in clearing brain Aβ (Wang, Wang, Zhang, et al. 2017; Liu et al. 2015).

#### 4.2.3 Therapies Based on Reducing Tau Phosphorylation and Misfolding and Microtubule Stabilization in AD and Other Tauopathies

Work by Holzer et al. has developed small molecule inhibitors of three kinases: GSK‐3β, Cdk5, and Cdk1 that are related to tau pathology. These new agents were able to show selectivity and potency towards the corresponding kinases and significantly reduced tau phosphorylation (Holzer et al. 2018). Giacomini et al. developed a small molecule inhibitor to TAOKs (Thousand‐and‐One Amino Acid Kinases), which are recently identified to be involved in tau phosphorylation and are activated at the tangles of AD and FTD brains. This TAOK inhibitor reduced aberrant tau phosphorylation in induced pluripotent stem cell‐derived neurons (iPSCs) from FTLD patients, as well as cortical neurons from a transgenic mouse model of tauopathy (Giacomini et al. 2018). Other potential drug candidates have been recently screened for their efficacy to reduce tau phosphorylation in vitro, such as Roscovitine (CDK5 inhibitor), Saracatinib (Src/Fyn kinase inhibitor), TBB (a casein kinase II inhibitor) and three GSK‐3 small molecule kinase inhibitors (Lithium Chloride, AR, and A‐107) (Yadikar et al. 2020). However, a phase IIa randomized clinical trial of Saracatinib in mild AD patients showed no significant effects of the drug on either CSF total tau or pTau (van Dyck et al. 2019).

Another strategy is to enhance tau elimination using passive and active tau immunotherapies (Table 1). Clinical trials of some anti‐tau humanized monoclonal antibodies are still ongoing to assess their safety and immunogenicity in patients with AD and other tauopathies (Table 1). The following mAb have successfully progressed to phase 2 clinical trials: JNJ‐63733657, which has high affinity to pThr217 (Galpern et al. 2019), and Bepranemab (UCB0107), which attacks the mid‐region of tau (amino acids 235–250) and can achieve 100% inhibition of fibrillization and aggregation and block pathogenic tau seeding in vivo (Albert et al. 2019). E2814, which recognizes an epitope in the microtubule‐binding domain near the mid‐domain of tau, and Lu AF87908, which targets pSer396 and pSer404, are still at early phases of development to assess their safety in healthy participants and those with MCI and mild AD. Nevertheless, the development of Zagotenemab, Semorinemab, Gosuranemab, and Tilavonemab, which can bind soluble tau aggregates at the N‐terminus, has been discontinued following the antibodies' failure in Phase II trials (Mullard 2021). Also, trials on PNT001 and RG7345, which target phospho‐tau epitopes, have terminated due to non‐safety‐related reasons and inflammatory responses (Song et al. 2022). Generally speaking, anti‐tau antibodies targeting the mid‐region of tau proved to be most effective against human tau pathologic seeds (from AD and PSP) than those targeting the N‐terminus region (Courade et al. 2018).

**TABLE 1:** List of ongoing monoclonal antibodies, vaccines, gene therapy‐based, and small molecules currently being tested in clinical trials for AD and FTD.

Therapy	Target	Delivery route	Sponsor	Phase	Clinical trial identifier/dates	Outcome	Status
*Alzheimer's disease*							
*Aβ‐targeted vaccines and monoclonal antibodies*							
Lecanemab (*LEQEMBI*, BAN2401)	Humanized IgG1 monoclonal antibody against soluble Aβ protofibrils	IV infusion/SC	Eisai and Biogen	II	NCT01767311 (2012–2024)	‐> To evaluate efficacy and safety in mild MCI and mild AD (3 dose levels: 2.5, 5, and 10 mg/kg) are given biweekly (once every 2 weeks) to separate groups of participants, and 2 dose levels (5 and 10 mg/kg) are given monthly (once every 4 weeks) to separate groups of participants	Active, not recruiting
				III	NCT03887455 (2019–2027)	‐> To evaluate long‐term efficacy and safety in early AD patients using Clinical Dementia Rating‐Sum of Boxes (CDR‐SB)	Active, not recruiting
				III	NCT04468659 (2020–2031) (AHEAD 3–45 Study)	‐> To evaluate the efficacy and safety of Lecanemab in preclinical AD and elevated amyloid and in patients with early preclinical AD and intermediate amyloid	Recruiting
Donanemab (LY3002813)	Humanized IgG1 monoclonal antibody that recognizes a modified form of Aβ deposits in the plaques (N‐truncated pyroglutamate form of Aβ (p3‐42).	IV	Eli Lilly and Company	II	NCT03367403 (2017–2021)	‐> Significant slowed cognitive and functional decline/better score of cognition and abilities to perform daily activities	Completed
				III	NCT04437511 (2020–2025)	‐> Early symptomatic AD (prodromal AD and mild dementia due to AD) with the presence of brain tau pathology	Active, not recruiting
Remternetug (LY3372993)	Humanized IgG1 monoclonal antibody that binds to the N‐terminal of pyroglutamated form of Aβ	IV	Eli Lilly and Company	I	NCT04451408 (2020–2024)	‐> Evaluate the safety and tolerability of LY3372993 in participants with AD, non‐Japanese, and healthy Japanese participants of first‐generation Japanese origin	Active, not recruiting
				I	NCT03720548 (2018–2019)	‐> Evaluate the safety and tolerability of LY3372993 in both healthy and AD participants ‐> Investigate how much LY3372993 gets into the bloodstream ‐> Test the effects of LY3372993 in participants with AD	Completed
				III	NCT06653153 (2024–2030)	‐> Early AD participants at risk for cognitive and functional decline	Recruiting
				III	NCT05463731 (2022–2026)	‐> Evaluate the safety and efficacy in participants with early symptomatic AD	Active, not recruiting
			Washington University School of Medicine	II/III	NCT06647498 (2024–2034)	‐> Determine its effectiveness in the treatment of asymptomatic AD (at risk) or patients with a type of Early Onset AD caused by a genetic mutation	Recruiting
Trontinemab (RO7126209)	A new version of the anti‐amyloid monoclonal antibody gantenerumab, designed to more easily cross the BBB using “brain shuttle” technology. A Fab fragment is attached to its Fc domain binds to human transferrin receptors on the brain endothelial cells leading to endocytosis and release into the brain parenchyma	IV infusion	Hoffmann‐La Roche	I	NCT04023994 (2019–2020)	‐> A single ascending dose administered to healthy participants is safe and tolerated with no hematology‐related adverse effects and showed increased uptake into the brain and more homogenous distribution	Completed
				Ib/IIa	NCT04639050 (2021–2028)	‐> To investigate the safety, tolerability, pharmacokinetics, and pharmacodynamics of multiple ascending doses of RO7126209 (given once every 4 weeks) in participants with prodromal or mild to moderate AD	Recruiting
PRX012	A humanized monoclonal IgG1 antibody to an N‐terminal epitope on Aβ with 10‐fold greater binding affinity for Aβ fibrils than Aducanumab and 20‐fold higher than Lecanemab and more potency than Donanemab	SC	Prothena	I			
ACU193 (Sabirnetug)	Humanized IgG2 monoclonal antibody that selectively targets toxic soluble amyloid‐β oligomers (the most neurotoxic form)	IV or SC	Acumen Pharmaceuticals	I	NCT06511570 (6–9/2024)	To assess the safety, tolerability, and pharmacokinetics of single‐dose IV ACU193 and multiple‐dose SC ACU193+rhuph20 (recombinant human hyaluronidase) in healthy participants	Completed
				I	NCT04931459 (2021–2023) (INTERCEPT‐AD)	To evaluate the safety, tolerability, and blood levels of single and multiple doses of ACU193 in patients with MCI or mild AD	Completed
				II	NCT06335173 (2024–2026) (ALTITUDE‐AD)	To evaluate the efficacy and safety of IV ACU193 infusion (once every 4 weeks) in early AD	Recruiting
ACI‐24	Liposome vaccine‐based array of Aβ1–15 sequences	SC	AC Immune SA	I	NCT02738450 (2016–2021)	Favorable safety and tolerability	Completed
				II	NCT04373616 (2021)	Adults with Down syndrome	Withdrawn
UB‐311	Synthetic Aβ vaccine with two N‐terminus Aβ1–14–targeting peptides coupled with a helper T‐cell epitope	IM	United Neuroscience Ltd.	II	NCT02551809 (2015–2018)	Safe, well‐tolerated, and generated a robust immune response and cognitive improvement in mild AD patients	Completed
ABvac40	Multiple repeats of a short C‐terminal fragment of Aβ40 coupled to KLH. Targeted against the C‐terminal end of Aβ40	SC	Araclon Biotech S.L.	I	NCT03113812 (2014–2015)	Safe and tolerable (no cases of vasogenic edema or microhemorrhages)	Completed
				II	NCT03461276 (2017–2023)	Amnestic MCI or very mild AD Results show a favorable safety and tolerability profile No incidents of vasogenic edema, sulcal effusion, or microhemorrhages were detected ABvac40 elicited a consistent and specific immune response	Completed
*Inhibitors of amyloid oligomers formation and aggregation*							
ALZ‐801 (Valiltramiprosate; valine‐conjugated prodrug of Tramiprosate)	Small molecule inhibitor of amyloid oligomer formation and aggregation	Oral	Alzheon Inc.	I	NCT04585347 (9–11/2015)	To assess the PK of a single oral dose of ALZ‐801 in healthy participants	Completed
				I	NCT04157712 (2015–2016)	To evaluate the safety, tolerability, and pharmacokinetics, of multiple doses of ALZ‐801 capsule formulation in healthy elderly subjects	Completed
				II	NCT04693520 (2020)	To investigate the effects of oral ALZ‐801, in Early AD subjects with APOEε4 gene variant (ApoE4/4 or ApoE3/4) carriers	Active
				III	NCT04770220 (2021–2024)	To evaluate the safety and efficacy of ALZ‐801 in patients with early AD and ApoE4/4 homozygotes The primary efficacy outcome assessment measures cognition (ADAS‐cog 13)	Completed
LM11A‐31	Small molecule p75^NTR^ ligand	Oral	PharmatrophiX	I/II	NCT03069014 (2017–2020)	To determine the safety of 2 doses of LM11A‐31‐BHS in patients with AD and to access biomarker and clinical exploratory endpoints	Completed
*Tau targeted vaccines and monoclonal antibodies*							
JNJ‐63733657	Humanized anti‐tau monoclonal Ab that targets pTau.	IV infusion	Janssen Research & Development LLC	I	NCT03375697 (2017–2019)	Healthy and AD subjects. Tolerated with dose‐dependent reductions in free p217 tau in CSF	Completed
				II	NCT04619420 (2021–2032)	To evaluate the effect of JNJ‐63733657 on clinical decline as measured by the Integrated Alzheimer's Disease Rating Scale (iADRS)	Active, not recruiting
Bepranemab (UCB0107)	Humanized monoclonal IgG4 Ab that binds to the central region of tau near tau's microtubule‐binding domain	IV infusion	Hoffmann‐La Roche, UCB S.A	I	NCT03464227 (02–12/2018)	‐> To study the safety and tolerability of a single ascending dose in healthy Male subjects	Completed
				II	NCT03605082 (2018–2019)	‐> To evaluate the safety and tolerability and serum Pharmacokinetics (PK) of single doses of UCB0107 in healthy Japanese subjects	Completed
				II	NCT04867616 (2021–2025)	‐> To study the safety and effect on the Clinical Dementia Rating Scale Sum of Boxes (CDR‐SB) in prodromal or mild AD	Active, not recruiting
E2814	Humanized, monoclonal IgG1 antibody that recognizes an epitope in the microtubule‐binding domain near the Tau mid‐domain	IV infusion	Eisai Inc.	I	NCT04231513 (2019–2023)	To assess safety, tolerability, PK, immunogenicity, and PD of IV infusions of E2814 in healthy subjects (combined single dose and multiple ascending dose study)	Completed
				Ib/II	NCT04971733 (2021–2024)	Mild to moderate cognitive impairment due to dominantly inherited AD	Completed
				II/III	NCT05269394 (Lecanemab+E2814) (2021–2028)	Early onset AD caused by genetic mutation (DIAN‐TU‐001 study)	Active, not recruiting
Lu AF87908	Humanized mouse IgG1 monoclonal antibody to pSer396 and pSer404	IV infusion	Lundbeck	I	NCT04149860 (2019–2023)	To investigate the safety of a single dose in healthy participants and patients with AD	Completed
BMS‐986446 (PRX005)	Anti‐tau IgG1 humanized antibody that recognizes an epitope in the R1, R2, and R3 repeats in the microtubule‐binding region which drives tau aggregation.	IV	Bristol‐Myers Squibb	I	NCT06084598 (2023–2024)	To evaluate the safety, tolerability and drug levels of a single IV dose of BMS‐986446 in healthy participants	Completed
				II	NCT06268886 (2024–2027)	To assess the effectiveness, safety, and tolerability in participants with Early AD	Recruiting
AADvac‐1	Synthetic peptide derived from tau amino acids sequence (DeMattos et al. 2002, 2001; Yamada et al. 2009; Liu et al. 2014; Budd Haeberlein et al. 2022; Morató et al. 2022; van Dyck et al. 2022; Larkin 2023; Reardon 2023; Harris 2023; Mintun et al. 2021; Wicker et al. 2024) coupled to KLH. Induces immune response against pathologically modified forms of tau protein	SC	Axon Neuroscience SE	I	NCT01850238 (2013–2015)	Safety and efficacy study in mild to moderate AD (3 months) → safe, well‐tolerated, immunogenic	Completed
				I	NCT02031198 (2014–2016)	Follow‐up safety study (18 months) for patients completed phase I previous study	Completed
				II	NCT02579252 (2016–2019)	Safety and efficacy study in mild AD (24 months) → significant reduction of CSF pTau217 and plasma levels of NfL and trend towards slower cognitive decline	Completed
				I	NCT03174886 (2017–2020)	Pilot Study in Patients with Non‐Fluent Primary Progressive Aphasia (nfvPPA) (24 months)	Unknown status
ACI‐35.030	Active liposome‐based anti‐pTau vaccine with an epitope to activate helper T cells	SC	AC Immune SA	Ib/IIa	NCT04445831 (2019–2023)	Double‐blind, randomized, placebo‐controlled study to evaluate the safety, and immunogenicity in early AD	Completed
IONIS‐MAPTRx (BIIB080)	Antisense oligonucleotide (ASO) targeting Tau expression	Intrathecal	Biogen, IONIS Pharmaceuticals Inc.	I/II	NCT03186989 (2017–2022)	Mild AD → safe with a dose‐dependent reduction in CSF pTau181 (phase I)	Completed
*Antioxidant and anti‐inflammatory approaches*							
REM0046127	Orai calcium (Ca^2+^) channel activity modulator	Oral	reMYND	I	NCT04672135 (2020–2022)	Study safety, tolerability, and PK in healthy subjects	Completed
				II	NCT05478031 (2022–2024)	In mild to moderate AD Terminated due to the event of repeated elevated transaminase levels in one subject up to 6× ULN in the absence of any other potential underlying cause	Terminated
Edaravone	Nrf2/HO‐1 pathway activator (antioxidant and neuroprotective)	Oral	Treeway B.V.	II	NCT05323812 (2023–2025)	Assess safety, PK and PD of oral Edaravone in mild AD	Active, not recruiting
Genistein	Phytoestrogen that has antioxidant, anti‐inflammatory and neuroprotective effects	Oral	Fundación para la Investigación del Hospital Clínico de Valencia	NA	NCT01982578 (2017–2020)	To determine the effect of 60 mg twice daily of genistein administration in AD patients. Results showed reduced amyloid deposits and a tendency towards cognitive function improvement	Completed
*Gene therapy*							
LX1001	AAV gene transfer vector expressing the cDNA coding of human APOE2 protein	Intrathecal	Lexeo Therapeutics	I/II	NCT03634007 (2019–2024)	In APOE4 Homozygote AD patients. Initial results indicated no adverse effects and decline in total and pTau	Completed
AAV2‐BDNF (Growth Factor Gene Therapy)	AAV‐2 expressing the human BDNF cDNA	MRI‐guided stereotaxic brain injection	Mark Tuszynski Ohio State University	I	NCT05040217 (2022–2027)	To test whether brain administration of AAV2‐BDNF Gene will slow or prevent cell loss in the brains of AD patients or those with MCI	Recruiting
*Therapies targeting insulin resistance*							
Metformin	Antidiabetic that reduces peripheral hyperinsulinemia and enhances insulin sensitivity by suppressing hepatic glucose production, increasing peripheral glucose uptake, and decreasing fatty acid oxidation leading to reduced pancreatic insulin secretion	Oral	Columbia University	II	NCT00620191 (2008–2012)	To study the effect of metformin in Amnestic MCI. Results were in favor of metformin vs. placebo regarding the Selective Reminding Test (SRT) and the score of the Alzheimer's Disease Assessment Scale‐cognitive subscale (ADAS‐cog). However, differences were not statistically significant possibly due to the small sample size.	Completed
			University of Pennsylvania	II	NCT01965756 (2013–2017)	To study the effect of metformin on AD biomarkers in non‐diabetic older adults with MCI. Metformin treatment was associated with enhanced executive functioning and suggested improvement in learning/memory and attention	Completed
			Columbia University	II/III	NCT04098666 (2021–2027)	To assess the efficacy of long‐acting metformin in AD prevention in patients with early and late MCI, without diabetes, overweight or obese, aged 55 to 90 years	Active, not recruiting
Liraglutide	GLP‐1 receptor agonist that reduces insulin resistance by inducing glucose‐dependent insulin secretion, suppresses hepatic gluconeogenesis and glucagon release	SC	Imperial College London	II	NCT01843075 (2014–2019)	To study its effect in mild AD	Unknown status
			University of Aarhus	NA	NCT01469351 (2012–2013)	To study its potential effects on degenerative changes in AD	Completed
			Stanford University	I	NCT02140983 (2013–2017)	To study the effects of Liraglutide on hippocampal structure and function in aging adults with prediabetes	Completed
Semaglutide	GLP‐1 receptor agonist	SC/Oral	Novo Nordisk A/S	III	NCT05891496 (2023–2025)	To study the effect of Semaglutide (SC once weekly) on the immune system and other biological processes in people with AD	Active, not recruiting
				III	NCT04777396 (2021–2026)	To study the safety and efficacy of oral Semaglutide in early AD subjects (EVOKE).	Active, not recruiting
				III	NCT04777409 (2021–2026)	To study the effect and safety of oral Semaglutide in early AD subjects (EVOKE Plus)	Active, not recruiting
			Rutgers, The State University of New Jersey	II	NCT06072963 (2024–2028) (COMMETS)	To study the effect of intranasal Insulin with oral Semaglutide to improve glucose uptake, cognition, cerebral blood flow and related blood biomarkers in older adults with metabolic syndrome and MCI. (COMMETS‐Combination MCI Metabolic Syndrome)	Recruiting
Liraglutide + Empagliflozin + Linagliptin	GLP‐1 receptor agonist/SGLT‐2 inhibitor/DPP‐4 inhibitor	SC/Oral	The Affiliated Nanjing Drum Tower Hospital of Nanjing University Medical School	NA	NCT05313529 (2022–2027)	To evaluate the effects of this combination in MCI remission in patients with type 2 diabetes. (LIGHT‐MCI)	Recruiting
Insulin		Intranasal (IN)	University of Southern California	II/III	NCT01767909 (2014–2018) (SNIFF)	‐>To examine the effects of IN‐administered insulin on cognition, entorhinal cortex and hippocampal atrophy, and CSF biomarkers in amnestic MCI or mild AD over a 12‐month period ‐>No cognitive or functional benefits were observed	Completed
		IN	Health Partners Institute	II	NCT01436045 (2011–2013)	‐>To investigate the effect of the rapidly acting IN insulin derivative (Glulisine) on memory and cognition in mild–moderate AD ‐>No significant effect on cognitive outcome	Completed
		IN	Beth Israel Deaconess Medical Center	II	NCT02415556 (2015–2020) (MemAID)	‐>To determine the long‐term effects of IN insulin on cognition and memory in older type 2 diabetic and non‐diabetic groups ‐>Results showed positive effects on cognition and gait, increased cerebral blood flow and lower plasma insulin and insulin resistance	Completed
		IN	Wake Forest University Health Sciences	II	NCT01595646 (2011–2015)	‐>To examine the effects of IN long‐acting insulin detemir on cognition in patients with AD or amnestic MCI ‐>Results showed significant improvement of verbal working memory and visuospatial working memory but no differences were found for daily functioning or executive functioning	Completed
		IN	University of Washington	II	NCT00438568 (2006–2011) (SNIFF 120)	‐>To study the effect of IN in AD or MCI for 4 months	Completed
		IN	Wake Forest University Health Sciences	I	NCT02462161 (2015–2019) (SNIFF‐Quick)	‐>To examine the effects of IN short‐acting Insulin Aspart on cognition, blood and CSF markers of AD, and Aβ deposition in the brain for 12‐week treatment period	Completed
		IN	University of Washington	II	NCT01547169 (2011–2012) (SNIFF‐LONG 21)	‐>To study the effects of IN‐administered long‐acting insulin detemir on cognition in persons with AD or amnestic MCI for 21‐day treatment period	Completed
		IN	Wake Forest University Health Sciences	II	NCT05006599 (2025–2031) (SNIFF—3‐Week Aptar CPS Device)	To examine if patients with preclinical AD can administer IN insulin (Humulin R U‐100) using delivery device four times daily over a 4 week period and will assess its impact on cognition, CSF biomarkers, and cerebral perfusion	Not yet recruiting
Insulin + Empagliflozin		IN/oral	Wake Forest University Health Sciences	II	NCT05081219 (2021–2024) SNIFF—Combo INI+EMPA Trial	‐>To assess safety and efficacy of either singular and combined effects of IN insulin (Humulin R U‐100) and Empagliflozin, to correct bioenergetic and vascular dysfunction in adults with preclinical or early AD and amnestic MCI over a 4‐week treatment period	Completed
*Recent approaches*							
Icapamespib (PU‐AD)	Epichaperomes Inhibitors	Oral	Samus Therapeutics	I	NCT03935568 (2019)	To evaluate safety and PK in healthy volunteers → generally safe and well‐tolerated following single or multiple doses up to 30 mg. Headache is the most common reported adverse event	Terminated due to the company ceased operations
				II	NCT04311515 (2020)	Mild AD	Terminated (company closure)
				II	NCT04505358 (2023)	ALS	Withdrawn
Varoglutamstat (PQ912)	Small molecule inhibitor of the glutaminyl cyclase enzyme	Oral	Vivoryon Therapeutics N.V.	II	NCT03919162 (2021–2024)	To evaluate the efficacy and safety in patients with early AD (VIVA‐MIND)	Terminated not due to safety concerns
				II	NCT04498650 (2020–2024)	To evaluate safety, tolerability and efficacy in subjects with MCI and mild AD	Completed
*Frontotemporal dementia*							
*Tau‐targeting therapies*							
AADvac1	Active Tau vaccine	SC	Axon Neuroscience SE	I	NCT03174886 (2017–2020)	Assess safety and immunogenicity in non‐fluent variant of Primary Progressive Aphasia (nfvPPA)	Unknown status
Bepranemab (UCB0107)	Humanized, monoclonal IgG4 antibody against the central region of tau	IV infusion	Hoffmann‐La Roche, UCB S.A.	I	NCT04185415 (2019–2021) NCT04658199 (2020–2027)	‐> Patients with PSP (proved to be safe and well‐tolerated with no evidence of anti‐drug antibodies) ‐> To assess safety and tolerability of long‐term administration in Progressive Supranuclear Palsy (PSP)	Completed Active, not recruiting
Fasudil	Rho‐associated protein kinase inhibitor	IV	Paul Lingor, Technical University of Munich	II	NCT03792490 (2019–2023)	‐> Analyze the safety, tolerability and efficacy of Fasudil in ALS	Completed
NIO752	Antisense oligonucleotide to Tau that interferes with Tau mRNA translation.	Intrathecal	Novartis Pharmaceuticals	I	NCT04539041 (2021–2024)	‐> Assess safety, tolerability, and PK in PSP (multiple dose escalation study)	Completed
				I	NCT05469360 (2023–2025)	‐> To assess the pharmacodynamics, safety, tolerability, and PK of NIO752 in patients with early AD	Active, not recruiting
RT001	A synthetic linoleic acid (LA) that inhibits lipid peroxidation	Oral	Retrotope	II	NCT04762589 (2021)	‐> ALS	Unknown status
				II	NCT04937530 (2021)	‐> PSP	
Progranulin (PGRN)‐targeting therapies and *C9orf72*‐associated ALS							
AZP2006	A small molecule that binds prosaposin binds to prosaposin, a cofactor for progranulin processing, and stabilizes the prosaposin‐progranulin complex preventing progranulin cleavage and increasing its secretion.	Oral	AlzProtect SAS	I	NCT04008355 (2020–2024)	‐> Healthy subjects → No safety issues	Completed
				II		‐> To assess tolerability, safety, PK, and effect of AZP2006 at different doses on CSF biomarkers in patients with PSP	Active, not recruiting
AL001 (Latozinemab)	Recombinant human anti‐sortilin (SORT1) monoclonal IgG1	IV infusion	Alzamend Neuro Inc.	I	NCT03636204 (2018–2019)	Healthy volunteers and FTD‐*GRN* mutation carriers (to assess safety, tolerability, PK and PD) → Safe, well‐tolerated and dose‐dependent increase in PGRN levels	Completed
				I/II	NCT05363293 (2022–2023)	Mild to moderate AD patients and healthy adult subjects (multiple ascending dose trials to determine the safety and maximum tolerated dose).	Completed
				II	NCT05053035 (2021–2022)	*C9orf72*‐associated ALS. A subsequent study is considered with different inclusion/exclusion criteria	Terminated
				II	NCT03987295 (2019–2026)	Evaluate the safety of long‐term AL001 dosing in FTD‐*GRN* or ‐*C9orf72* associated gene mutations	Active, not recruiting
				III	NCT04374136 (2020–2027)	Evaluating the efficacy and safety of AL001 in participants at risk for or with FTD due to heterozygous mutations in the PGRN gene (INFRONT‐3)	Active, not recruiting
PR006A (LY3884963) PBFT02	AAV1 vectors of functional *GRN* gene	Intrathecal injection into the Cisterna Magna	Eli Lilly & Co., Prevail Therapeutics	I/II	NCT04408625 (2020–2029)	FTD With PGRN mutations	Recruiting
			Passage Bio	I/II	NCT04747431 (2021–2027)	To assess the safety, tolerability, and PD of a single dose of PBFT02 in patients with FTD‐*GRN* gene mutations	Recruiting
Metformin	Antidiabetic drug to reduce the expression of toxic repetitive proteins produced from the *C9orf72* repeat expansion	Oral	University of Florida	II	NCT04220021 (2020–2025)	To assess the safety and potential efficacy of Metformin in *C9orf72‐*ALS/FTD	Active, not recruiting
TPN‐101 (Censavudin)	Reverse‐transcriptase inhibitor that inhibits the reactivation and expression of LINE‐1	Oral	Transposon Therapeutics Inc.	IIa	NCT04993755 (2021–2023)	To assess the safety and potential efficacy of Metformin in C9orf72‐ALS/FTD	Active, not recruiting
				IIa	NCT04993768 (2021–2023)	To assess safety in PSP	Active, not recruiting

Results from a Phase I clinical study of subcutaneous injections of AADvac‐1 active immunization in mild to moderate AD patients indicated overall safety and tolerability and that it is highly immunogenic (Novak et al. 2018). Phase II trials showed that AADvac‐1 significantly reduced CSF pTau217 and plasma levels of neurofilament light‐chain protein (NfL), an emerging biomarker of neurodegeneration that correlates with neurofibrillary pathology in the brain. Although the study did not show any significant effect on cognitive and functional tests overall, post hoc analysis revealed that the vaccine slowed down cognitive and functional decline in AD patients having both amyloid and tau pathology and attenuated white matter degeneration (Novak et al. 2021). Therefore, further studies are required to confirm its clinical efficacy and impact on disease biomarkers. There is also an ongoing Phase I pilot study of AADvac‐1 in patients with non‐fluent primary progressive aphasia (nfvPPA) that is caused by tau pathology. The study is primarily assessing safety and exploring any potential efficacy in this type of FTD by measuring CSF biomarkers, such as NfL and tau, as well as evaluating the effects of treatment on language impairment, behavior, cognition, and motor function. ACI‐35.030 is another active liposome‐based anti‐phospho‐tau vaccine. It is currently under Phase 1b/2a clinical trials to assess safety, tolerability, and immunogenicity in patients with early AD and will also monitor cognition and behavioral changes among participants. Early data show that ACI‐35.030 treatment is safe and leads to a strong and durable immunogenic response (AC Immune 2022). The use of small interfering RNAs (siRNAs) to reduce tau expression proved to be effective in animal models of tauopathies. A different strategy is to prevent tau aggregation via reduction of tau protein levels using antisense oligonucleotides (ASOs). Phase I clinical studies with ASOs in patients with mild AD were recently completed. First results revealed no serious adverse events following intrathecal injections of multiple doses and a dose‐dependent reduction in total tau and pTau‐181 in CSF (Rösler et al. 2019; Mummery and Junge 2021). Nusinersen, an ASO drug that modulates pre‐messenger RNA splicing, has already been approved for clinical use in spinal muscular atrophy (Mercuri et al. 2018). It is also worth mentioning that microtubule stabilizing agents such as TPI‐287 (abeotaxane), Epothilone D (Zhang et al. 2012) and NAP (davunetide) (Quraishe et al. 2013) have been introduced as potential drug candidates for tauopathies (Fernandez‐Valenzuela et al. 2020); however, clinical trials indicated less tolerability to TPI‐287 in AD patients owing to hypersensitivity reactions, and clinical worsening was observed in those with progressive supranuclear palsy (PSP) and corticobasal syndrome (CS) (Tsai et al. 2020) (NCT019666666 and NCT02133846).

#### 4.2.4 Oxidative Stress‐Based Therapies, Anti‐Inflammatory, and Neuroprotective Approaches

Therapeutic approaches including vitamins (vitamin E, C, and B_12_), flavonoids (curcumin, resveratrol and genistein) and active antioxidants (selenium, selegiline and α‐lipoic acid) represent a hopeful strategy to delay or prevent the disease progression. Several studies have reported promising results of antioxidants in AD animal models in terms of neuroprotection, antioxidant, and anti‐inflammatory effects (Nadeau and Roberge 1988; Rajasekar et al. 2013; Sung et al. 2004); however, results from randomized clinical trials are not consistent and inconclusive (Ringman et al. 2012; Galasko et al. 2012; Sano et al. 1997; Pavlik et al. 2009). Administration of cocktail antioxidants combined with traditional therapies may be more effective than when the antioxidant is given alone (Kamat et al. 2016). An example of that is a natural product mixture composed of docosahexaenoic acid, *Ginkgo biloba* , D‐pinitol, and ursolic acid, which displayed synergistic neuroprotective effects in an AD murine model (Ferre et al. 2021). Developing compounds that can cross the BBB and directly target the mitochondria, such as Coenzyme Q, may be promising. Curcumin has been reported to have potential benefits in cognition and is effective in reducing Aβ and tau aggregation besides its multifaceted actions as antioxidant, anti‐inflammatory, anti‐angiogenic, and neuroprotective. Studies showed that Curcumin stimulates Aβ uptake and phagocytosis by microglia/macrophages (Zhang et al. 2006) and inhibits the JNK signaling pathway (Chen and Tan 1998). Clinical studies are ongoing to assess the efficacy and tolerability of a number of novel curcumin formulations and analogues with enhanced oral bioavailability (Soeda and Takashima 2020). Another compound, *scyllo*‐Inositol (one of the stereoisomers of Inositol) is a naturally occurring compound that can cross the BBB and has been shown to inhibit Aβ aggregation and break up oligomers (Lee et al. 2017). Baicalein (BE) is a natural active flavonoid compound that has neuroprotective properties and a potential therapeutic benefit in neurodegenerative disorders, including AD and Parkinson's disease, owing to its antioxidant, anti‐inflammatory, and antiapoptotic effects (Sowndhararajan et al. 2017). Intranasal BE‐micellar formulation (Zhang et al. 2020) and intraperitoneal nanocarriers of BE were designed (Aghajanzadeh et al. 2020), and these formulations were able to achieve higher bioavailability and higher brain delivery and distribution of BE; thus, they could represent potential drug delivery systems for the treatment of neurodegenerative disorders. Genistein is a phytoestrogen found in soy that has antioxidant and antiapoptotic effects and was found to increase the expression of PPARγ receptors. Results showed that it may have a role in delaying the onset of dementia in patients with prodromal AD (Viña et al. 2022, 2024).

Other therapeutic strategies have been investigated, and some have demonstrated beneficial effects in clinical trials through their anti‐inflammatory and neuroprotective effects, besides reducing the activity of BACE and γ‐secretases, such as non‐steroidal anti‐inflammatory drugs (NSAIDs), anti‐TNF‐α, Peroxisome proliferators activated receptor (PPARγ) agonists (Jiang et al. 2012) and recently, repeated menthol inhalation improved cognitive function of AD mice through its immunomodulatory mechanisms and downregulation of IL‐1β and IL‐6 mRNA in the prefrontal cortex (Casares et al. 2023). A recent Phase 2a clinical trial is evaluating the PK/PD (pharmacokinetics/pharmacodynamics), safety, and tolerability of an oral AD drug candidate, REM0046127, in patients with mild to moderate AD. REM0046127 is a small molecule that acts through modulating ORAI Ca^2+^ channel activity and Ca^2+^ influx to normalize neuronal Ca^2+^ homeostasis. This mechanism of action is likely expected to restore synaptic plasticity and to slow amyloid plaque formation and neuronal cell death.

Non‐pharmacological treatments and lifestyle interventions such as regular physical activity and caloric restriction have also gained much attention due to their benefits on health and life span and improving cognitive function specifically with prediabetic individuals (Ntsapi and Loos 2016; Baker et al. 2010).

##### 4.2.4.1 Edaravone

Edaravone (3‐methyl‐1‐phenyl‐2‐pyrazolin‐5‐one, MCI‐186) is a potent free radical scavenger and antioxidant developed by Mitsubishi Tanabe Pharma Corporation (Osaka, Japan). It is a white to yellowish‐white crystalline powder that is freely soluble in acetic acid, methanol, or ethanol, while poorly soluble in water or diethyl ether (Cruz 2018). The lipophilic moieties in Edaravone (phenyl and methyl groups) render it able to penetrate the BBB (Watanabe et al. 2008).

Edaravone may act by reducing the oxidative injury in neurons and eliminating free radicals and lipid peroxidation products (Watanabe et al. 2018). Studies showed that Edaravone attenuates cell apoptosis and protects the stability and integrity of the BBB through activation of the Nrf2/HO‐1 pathway (the nuclear factor erythroid 2‐related factor/hemeoxygenase‐1) (Pan et al. 2020; Liu et al. 2019). Researchers also found that Edaravone may exert neuroprotective effects through suppressing gene expression of the Fas signaling pathway following ischemia in rats (Xiao et al. 2007; Li et al. 2018), reducing glial cell overactivation, and alleviating synaptic damage (Zhao et al. 2022). Edaravone may also exert antiapoptotic effects by blocking cytosolic release of cytochrome *c* and caspase‐3 activation in hypoxic–ischemic (HI) brain injury in neonatal rats (Yasuoka et al. 2004). It also reportedly upregulated Bcl‐2 and downregulated Bax expression in a transient focal ischemia model in rats (Amemiya et al. 2005).

Currently, Edaravone is approved for the treatment of acute ischemic stroke in Japan and other Asian countries and for the treatment of amyotrophic lateral sclerosis (ALS) by the FDA under the name of Radicava, provided as an intravenous infusion. Clinical studies showed that EDR reduces neuronal damage and suppresses proinflammatory responses following brain ischemia (Feng et al. 2011; Yoshida et al. 2006) and inhibits motor functional deterioration in ALS patients (Yoshino 2019). However, the main drawback for Edaravone use is that it requires administration via IV infusion on a regular basis, which is inconvenient and challenging for patients with progressive degenerative disorders. In this context, RADICAVA ORS (Edaravone oral suspension) was developed by Mitsubishi Tanabe Pharma Corporation America and approved by the FDA in December 2022 for the treatment of ALS. In addition, a Phase III clinical trial is ongoing to assess the efficacy and safety of two dosing regimens of oral Edaravone administration in subjects with ALS (ClinicalTrials.gov Identifier: NCT04569084). Also, another clinical trial is currently ongoing to assess safety, pharmacokinetics, and pharmacodynamics of oral Edaravone in mild AD.

Edaravone is considered the first disease‐modifying therapy to be approved and holds great promise to alleviate several other neurodegenerative diseases by targeting multiple key pathways of the disease pathogenesis. In this respect, animal studies showed that treatment of APP/PS1 AD mice with Edaravone intraperitoneal injection either before or after the onset of Aβ disposition reduced the plaque load in the brain, alleviated OS, and reduced downstream pathologies including neuroinflammation, synaptic dysfunction, and apoptosis, and ameliorated cognitive deficits in AD mice (Jiao, Yao, et al. 2015). Edaravone reduced tau phosphorylation through inhibiting GSK‐3β activity in AD mice. Studies also reported that Edaravone suppressed the fibrillation of Aβ in vitro and induced dissociation of the preformed Aβ fibrils. Furthermore, Edaravone protected human neuroblastoma‐derived neurons (SH‐SY5Y cells) against Aβ‐induced neurotoxicity and neurite collapse (Jiao, Yao, et al. 2015).

An oral Edaravone formulation designed by Parikh and colleagues (Parikh, Kathawala, Tan, et al. 2018; Parikh, Kathawala, Li, et al. 2018) using Soluplus as a carrier and a self‐nano micellizing solid dispersion strategy was able to dose‐dependently reverse the cognitive deficits and mood disorders of very old APP/PS1 mice (17 months). Oral Edaravone treatment improved sensorimotor functions and significantly reduced cerebral infarction in the middle cerebral artery occlusion model in rats (Zhao et al. 2022). Another study reported that Edaravone oral treatment alleviated cognitive and motor deficits and reduced OS parameters and tau phosphorylation in aged P301L mice (FTD‐tau animal model) (Kelliny et al. 2021).

Recent studies also suggest that Edaravone is effective in preventing end‐organ damage with diabetes. Edaravone increased the number of corneal nerve fibers and protected against retinal damage in animal models of diabetic retinopathy by reduction of ROS, downregulation of NF‐κB p65 expression, besides its antiapoptotic effects (Wan et al. 2019; Yuan et al. 2014). It also prevented the development of nephropathy in diabetic rats (Varatharajan et al. 2016) and accelerated chronic wound healing when applied as a topical hydrogel in diabetic mice (Fan et al. 2019). Chronic oral administration of Edaravone has also demonstrated therapeutic potential to ameliorate diastolic dysfunction in a rat model of diabetic cardiomyopathy, possibly by reducing hyperglycemia and hyperlipidemia‐induced cardiomyocyte hypertrophy, apoptosis, and fibrosis. The therapeutic effects of Edaravone to alleviate OS in experimental cerebral ischemia/reperfusion (Yu, Wu, et al. 2020), neurologic deficits (Zhang et al. 2018), diabetic cardiomyopathy (Wang et al. 2022), and asthma (Pan et al. 2020) are achieved by upregulation of the Nrf2 signaling pathway, nicotinamide adenine dinucleotide phosphate quinone oxidoreductase, and heme oxygenase (HO‐1).

#### 4.2.5 Gene Therapy

LX1001 (AAVrh.10hAPOE2) is an adeno‐associated virus (AAV) gene transfer vector expressing the complementary deoxyribonucleic acid (cDNA) coding for human APOE2 that is being developed by Lexeo Therapeutics. It is designed as a one‐time treatment to deliver the APOE2 gene into the CNS for the treatment of APOE4‐associated AD. Initial data from the low‐dose cohort of the ongoing Phase 1/2 trials, assessing the safety of intrathecal LX1001 administration, showed that LX1001 relatively raised the protective levels of CSF APOE2 in AD patients with APOE4 homozygotes and reduced core AD biomarkers in CSF (T‐tau and P‐tau) with no serious adverse events reported.

Open‐label Phase I clinical trial of AAV2‐BDNF gene therapy is ongoing to assess the safety, tolerability, and preliminary efficacy of AAV2‐BDNF stereotaxic brain injection in early AD and Mild Cognitive Impairment (MCI).

#### 4.2.6 Targeting Insulin Resistance as a Potential Disease‐Modifying Therapy for AD and Other Neurodegenerative Disorders

The findings that support the link between insulin resistance and the increased risk of dementia have led the investigations to explore the impact of related strategies on the prevention and treatment of AD (Nowell et al. 2023; Nowell, Blunt et al. 2023). Preclinical studies showed that incretin mimetics, GLP‐1/GIP (glucagon‐like peptide 1 and glucose‐dependent insulinotropic polypeptide (GIP)) agonists display neuroprotective effects, reduce neuroinflammation, reduce tau phosphorylation and amyloid deposition, increase synaptic function, and improve memory formation through modulating blood glucose levels and restoring brain insulin signaling (Nowell, Blunt et al. 2023). Studies showed that treatment with metformin in non‐diabetic obese patients with MCI improved some aspects of cognition (Luchsinger et al. 2016; Weinberg et al. 2024; Rosell‐Díaz and Fernández‐Real 2023). Furthermore, a current clinical trial is investigating the effects of a combination of Liraglutide, Empagliflozin, and Linagliptin on cognitive functions in Type2 diabetic patients with MCI (Crook and Edison 2024). A recent clinical trial examined the possible benefits of a novel therapeutic approach using intranasal insulin on cognition, CSF biomarkers, and hippocampal/entorhinal atrophy in amnestic MCI or mild AD over a 12‐month period. However, no significant differences were observed between treatment arms for the primary outcome and secondary clinical outcomes including CSF Aβ42 and Aβ40, total tau protein, and tau p‐181, and the ratios of Aβ42 to Aβ40 and Aβ42 to total tau levels were unchanged as well as the blood glucose and hemoglobin A_1c_ values (Wadman 2012; Craft et al. 2020). Another clinical trial found that intranasal insulin improved cognition and gait in old people with or without type 2 diabetes (Novak et al. 2022; Galindo‐Mendez et al. 2020). Intranasal administration of long‐acting Insulin Detemir improved some aspects of memory in patients with mild MCI or early‐stage AD who are APOE‐ε4 carriers (Claxton et al. 2015). In the same context, further clinical studies are underway to examine the combined effect of Insulin with oral Semaglutide or oral Empagliflozin in older adults with metabolic syndrome and MCI (Davidy et al. 2024) (Table 1).

#### 4.2.7 Further Recent Approaches

##### 4.2.7.1 Epichaperomes Inhibitors

Icapamespib (PU‐AD or PU‐HZ151) is a small molecule purine analogue that selectively inhibits epichaperome activity. Epichaperomes act as scaffolding platforms of chaperones, heat shock proteins such as Hsp90, and other regulatory proteins that are formed under stress and pathological conditions leading to misfolding and protein–protein interactions causing alterations in brain cells and synaptic dysfunction (Digwal et al. 2022). They play a central role in the propagation of aberrant tau in AD, α‐synuclein in Parkinson's disease, and TDP‐43 and FUS in ALS (Carman et al. 2013). Therefore, disruption of the epichaperome complex network may promote the degradation of misfolded proteins and restoration of cellular functions, thus representing a rational therapeutic approach for the treatment of AD and other neurodegenerative disorders such as tauopathies (Inda et al. 2020). Administration of PU‐AD into AD transgenic mice restored long‐term memory to the levels of wildtype mice and significantly reduced pathologic tau levels (Inda et al. 2020). PU‐AD, given orally, achieved a high brain concentration and has proven to be safe in healthy adults (Silverman et al. 2022). However, further drug development for Phase II clinical trials in AD and ALS has ceased (Table 1).

##### 4.2.7.2 Glutaminyl Cyclase (QC) and Iso‐QC Enzymes Inhibitors

Varoglutamstat is a novel small molecule that appears to be a promising therapeutic agent in AD. It exerts dual action on Aß and inflammation by inhibiting glutaminyl cyclase (QC) and iso‐QC enzymes. QC and iso‐QC are involved in the post‐translational modification of Aẞ peptides into toxic pyroglutamate Aß and cytokine monocyte chemoattractant protein‐1 (CCL2) into neuroinflammatory pGlu‐CCL2, both of which are upregulated in AD (Feldman et al. 2024). Varoglutamstat works by inhibiting the formation of pyroglutamate amyloid‐beta (AB) peptides. These specific peptides are known to be synaptotoxic, proinflammatory, resistant to degradation, and capable of aggregating into toxic Aß oligomers (Jawhar et al. 2011). Unlike Donanemab, which also targets pyroglutamate Aẞ, Varoglutamstat works differently by not relying on microglial‐mediated plaque clearance. Donanemab's approach, though effective, carries a risk of ARIA, which can cause brain swelling or hemorrhages in some patients. Early clinical trials are currently investigating the safety and tolerability of different dosage regimens in early AD patients.

#### 4.2.8 Further Recent Approaches for Pharmacological Treatments for FTD

Molecule‐based therapies are being developed to treat the 3 major genetic subtypes of FTD: *C9orf75*, *GRN*, and *MAPT*. Tau‐targeting therapies, including active vaccines (AADvac1), antibodies (Bepranemab), and Rho‐associated protein kinase inhibitor (Fasudil), which acts through enhancing tau autophagy and reducing tau mRNA, as well as an intrathecal antisense oligonucleotide (NIO752) that acts by interfering with the translation of tau mRNA, are being evaluated in clinical trials for their efficacy and safety in PSP, CBD, and other tauopathies, including AD. RT001 is an isotopically stabilized, synthetic linoleic acid (LA) designed to inhibit lipid peroxidation and OS and prevent cellular and mitochondrial membrane damage. It showed a small clinical benefit in patients with PSP and severe ALS, but further trials are required on a larger number of patients to confirm its clinical benefit (Magrath Guimet et al. 2022). AZP2006 (Ezeprogind) is a new, promising treatment for AD and PSP. It is an orally available small molecule that crosses the BBB and was found to increase the levels of the neurotrophic factor progranulin (PGRN), reduce neuroinflammation and tau accumulation, and promote neuronal survival and synaptogenesis in preclinical models of AD and aging. Phase I trials in healthy volunteers showed no specific risks with single ascending doses of 3–500 mg (Verwaerde et al. 2024). It has now completed Phase 2 clinical trials in PSP and has received “orphan drug” designation in both Europe and the USA for PSP and is currently under development for AD (Callizot et al. 2021). Another strategy to increase PGRN levels is through blocking Sortilin (Lee et al. 2014). In this context, AL001 (Latozinemab) is a recombinant human anti‐sortilin (SORT1) monoclonal IgG1 that is currently under development for the treatment of FTD‐*GRN* patients and *C9orf72*‐associated ALS. Phase 2 clinical trials showed that chronic administration of AL001 led to a sustained increase in PGRN levels in FTD‐*GRN* patients (Huang et al. 2022). Gene therapy is another approach to enhance PGRN levels. Both PR006A and PBFT02 are AAV1 vectors intended to deliver a functional copy of the GRN gene to the brain that are currently being developed for the treatment of FTD‐*GRN* patients (Heckman et al. 2020). For ALS patients carrying *C9orf72* mutations, Metformin and TPN‐101, in addition to AL001, are currently being assessed for their clinical safety and potential efficacy. Metformin, a well‐tolerated antidiabetic drug, was able to reduce the expression of toxic repetitive proteins produced from the *C9orf72* repeat expansion by interfering with a key pathway and to mitigate disease features in a *C9orf72*‐ALS/FTD mouse model (Rosbash 2020). TPN‐101 (censavudine), a reverse‐transcriptase inhibitor originally developed for the treatment of HIV infection, is currently in Phase 2 clinical trials to test its safety and efficacy in PSP and ALS or FTD associated with *C9orf72* gene repeat expansion. TPN‐101 is thought to inhibit the reactivation and expression of long interspersed nuclear elements 1 (LINE‐1) and other retrotransposons, which are mobile acquired DNA elements in our genome that use a reverse‐transcriptase enzyme to self‐replicate and proliferate. The abnormal expression of these proteins is triggered by aging, TDP‐43, or toxic tau, initiating a cascade of events that lead to immune response, neuroinflammation, and cell death (Magrath Guimet et al. 2022). Recently, Locanabio is developing a novel gene therapy using CRISPR technology for the treatment of *C9orf72*‐ALS. The therapy developer reported that this CRISPR‐based gene therapy significantly reduced the accumulation of toxic RNA molecules resulting from *C9orf72* repeat mutations in cells and ALS mouse models without affecting the levels of healthy *C9orf72* RNA (Fang et al. 2022).

## 5 Conclusions

In this review, we have reviewed the molecular mechanisms as well as the current symptomatic treatments of AD and FTD with a focus on novel therapeutic approaches based on recent theories and findings. Dementia and AD are very common with aging and affect memory and learning. With the expansion of knowledge on the disease mechanisms and diagnostic biomarkers, new techniques have been developed such as Aβ and tau vaccines and antibodies, small molecule neurotrophin ligands, and gene therapy. On the other hand, symptoms of FTD are not only limited to memory impairments but are accompanied by a complex array of behavioral, neuropsychiatric, and motor disturbances that make it difficult for clinicians to diagnose. Various protein deposits have been identified that lead to neurodegeneration in FTD and are primarily caused by genetic mutations. Understanding the pathophysiology underlying FTD has allowed the design of new molecule‐based therapies that are showing promising results but are still in the early stages of clinical development.

## Author Contributions

Sally Kelliny: conceptualization, investigation, visualization, writing – original draft preparation. Larisa Bobrovskaya and Xin‐Fu Zhou: conceptualization, supervision, writing – review and editing.

## Conflicts of Interest

The authors declare no conflicts of interest.

### Peer Review

The peer review history for this article is available at https://www.webofscience.com/api/gateway/wos/peer‐review/10.1002/jnr.70046.

## Supporting information

Transparent Science Questionnaire for Authors

## Data Availability

The authors have nothing to report.
